# Development of ADPribosyl Ubiquitin Analogues to Study Enzymes Involved in Legionella Infection

**DOI:** 10.1002/chem.202004590

**Published:** 2020-12-23

**Authors:** Robbert Q. Kim, Mohit Misra, Alexis Gonzalez, Ines Tomašković, Donghyuk Shin, Hermann Schindelin, Dmitri V. Filippov, Huib Ovaa, Ivan Đikić, Gerbrand J. van der Heden van Noort

**Affiliations:** ^1^ Oncode Institute and Department of Cell and Chemical Biology Leiden University Medical Centre Einthovenweg 20 2333 ZC Leiden The Netherlands; ^2^ Institute of Biochemistry II Goethe University Faculty of Medicine Theodor-Stern-Kai 7 60590 Frankfurt am Main Germany; ^3^ Buchmann Institute for Molecular Life Sciences Goethe University Frankfurt, Riedberg Campus Max-von-Laue-Strasse 15 60438 Frankfurt am Main Germany; ^4^ Rudolf Virchow Center for Integrative and Translational Bioimaging University of Würzburg Josef-Schneider-Strasse 2 97080 Würzburg Germany; ^5^ Leiden Institute of Chemistry Leiden University Einsteinweg 55 2333 CC Leiden The Netherlands; ^6^ Current Address: Department of Nano-Bioengineering Incheon National University Academyro 119 22012 Incheon South Korea

**Keywords:** ADPribosylation, click chemistry, Legionella, post-translational modifications, ubiquitin

## Abstract

Legionnaires’ disease is caused by infection with the intracellularly replicating Gram‐negative bacterium *Legionella pneumophila*. This pathogen uses an unconventional way of ubiquitinating host proteins by generating a phosphoribosyl linkage between substrate proteins and ubiquitin by making use of an ADPribosylated ubiquitin (Ub^ADPr^) intermediate. The family of SidE effector enzymes that catalyze this reaction is counteracted by Legionella hydrolases, which are called Dups. This unusual ubiquitination process is important for Legionella proliferation and understanding these processes on a molecular level might prove invaluable in finding new treatments. Herein, a modular approach is used for the synthesis of triazole‐linked Ub^ADPr^, and analogues thereof, and their affinity towards the hydrolase DupA is determined and hydrolysis rates are compared to natively linked Ub^ADPr^. The inhibitory effects of modified Ub on the canonical eukaryotic E1‐enzyme Uba1 are investigated and rationalized in the context of a high‐resolution crystal structure reported herein. Finally, it is shown that synthetic Ub^ADPr^ analogues can be used to effectively pull‐down overexpressed DupA from cell lysate.

## Graphical abstract

**Modular modifications**: Analogues of ADPribosylated ubiquitin, including a nonhydrolyzable methylenebisphosphonate version, are constructed and subsequently applied to investigate (de‐)ubiquitinating enzymes involved in Legionella infection. These probes are used in the study of the bacterial effector DupA and the human ubiquitin activating E1 enzyme.

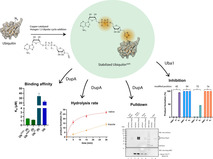

## Introduction

**Figure 1:**
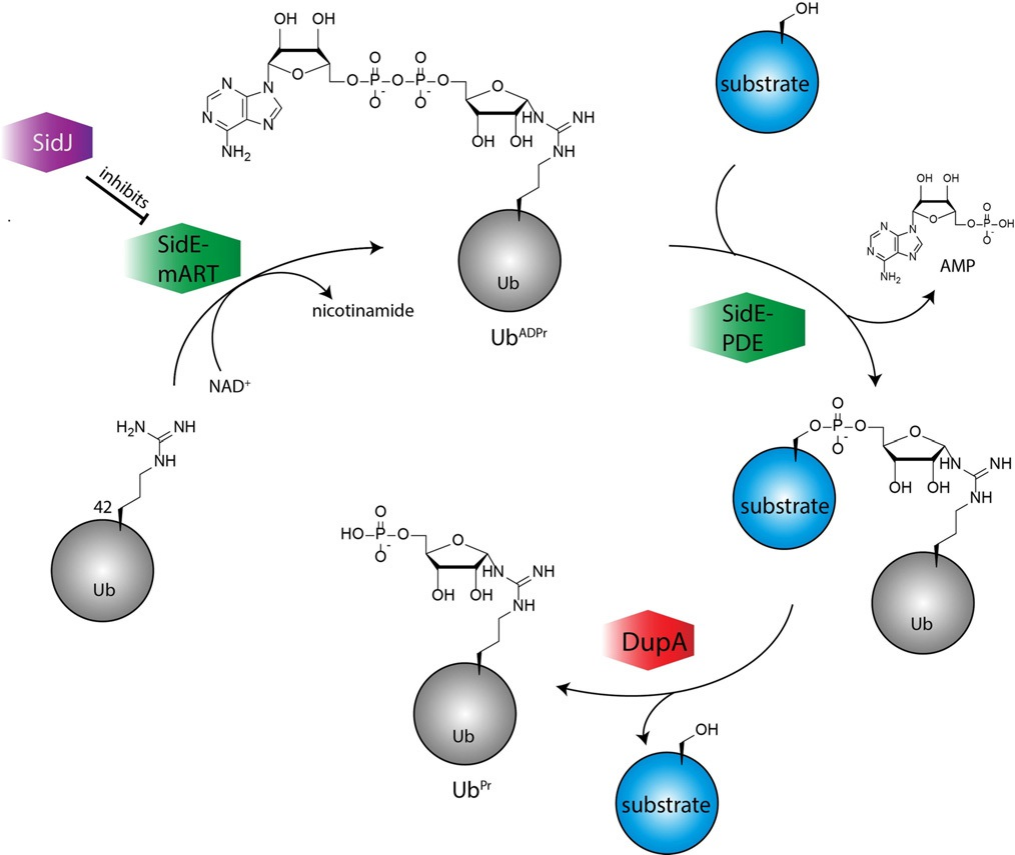
Schematic representation of substrate ubiquitination by noncanonical Legionella SidE enzymes and substrate release by DupA.

The dogma in post‐translational modification by ubiquitin (Ub) is that Ub‐activating enzymes (E1), Ub‐conjugating enzymes (E2), and Ub ligases (E3) are required to work together to activate the C‐terminal carboxylate of Ub, in an adenosine triphosphate (ATP)‐dependent manner, and subsequently ligate it to predominantly the ϵ‐amino group of a lysine in a substrate protein. Discovery of a class of *Legionella pneumophila* effector proteins that can conjugate Ub to substrate proteins, independent of the canonical machinery and without the need for ATP, has gained much interest.^[1]^ These multidomain bacterial enzymes are able to ADP‐ribosylate the δ‐guanidinium group of arginine 42 (Arg42) of Ub at the expense of nicotinamide adenine dinucleotide (NAD^+^) by using their mono‐ADP‐transferase (mART) domain in the first step, followed by the action of their phosphodiesterase (PDE) domain, which catalyzes the transfer of phosphoribose‐Ub (Ub^Pr^) to the serine of a substrate protein, while expelling adenosine monophosphate (AMP; Figure 1).^[2]^ Legionella has its own regulatory mechanism in place to control the temporal activity of these SidE ligases by blocking their active‐site glutamate using the glutamylase SidJ.^[3]^ The recently identified deubiquitinases for phosphoribosyl ubiquitination (Dups), DupA and DupB, also known as LaiE and LaiF, counteract the SidE‐mediated attachment of phosphoribosyl‐linked Ub to substrates.^[4]^ DupA and DupB were identified on the basis of their structural homology to the SidE PDE domains, but lack the ability to Pr‐ubiquitinate the substrate protein Rab33b upon incubation with ^Arg42^Ub^ADPr^. These DUPs, however, were shown to release proteins that were Pr‐ubiquitinated by SidE ligases by cleaving the phosphodiester bond between the substrate serine and ^Arg42^Ub^Pr^.^[4a]^ Although SidE ligases and Dups have opposite functions, they are structurally very similar and, even more so, the ligase SdeA is shown to mediate hydrolysis of the pyrophosphate bond in Ub^ADPr^ if no suitable substrate protein is present. The ligase effectively mediates transfer of a water molecule instead of a serine residue to the activated pyrophosphate bond, thereby expelling AMP.^[1b]^

By using a catalytically inactive version of DupA to enrich for Pr‐ubiquitinated substrates in HEK293T cells infected with Legionella, 180 host proteins were identified based on their affinity for DupA.^[4a]^ Most of these proteins are involved in endoplasmic reticulum membrane recruitment to Legionella‐containing vacuoles (LCVs). This highlights the importance of Pr‐ubiquitination upon Legionella infection because maintaining LCV integrity is essential for Legionella proliferation and the onset of Legionnaires’ disease.

**Figure 2:**
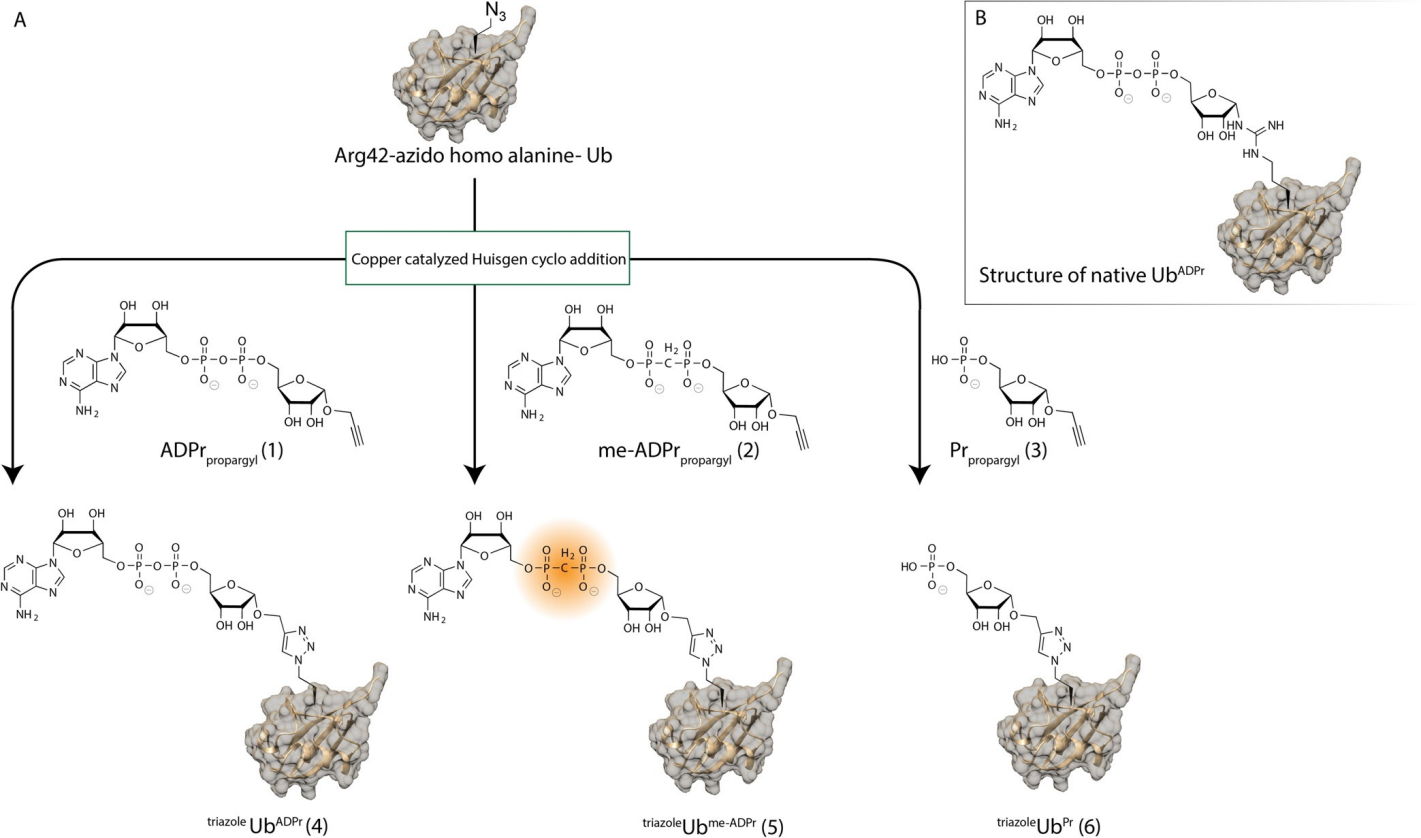
A) Modular approach of using click chemistry to construct ^triazole^Ub analogues. B) Schematic structure of native ^Arg^Ub^ADPr^.

In the canonical ubiquitination pathway, the use of chemically prepared tools, such as substrate reagents and activity‐based probes, has been a widely applied and successful approach to allow the study of kinetic parameters, as well as capturing and identifying both ligases and proteases.^[5]^ The recent development of fluorescent polarization based assay reagents and inhibitors to study enzymes involved in the Pr‐ubiquitination pathway highlights the applicability of chemically synthesized tools to study Pr‐ubiquitination.^[6]^ Hence, the construction of probes targeting the ADPr‐mediated ubiquitination machinery will be a similarly useful asset in studying the enzymes involved. We set out to prepare α‐*O*‐propargyl ADPr (**1**; Figure 2) and its stabilized methylene bisphosphonate analogue, α‐*O*‐propargyl me‐ADPr (**2**), in which oxygen in the pyrophosphate linkage is replaced with a methylene group.^[7]^ Facile copper‐catalyzed Huisgen azide‐to‐alkyne 1,3‐dipolar cycloaddition (CuAAC, or click reaction) of these propargyl‐containing ADPr analogues to azide‐modified Ub allowed the generation of probes **4** and **5** to investigate Legionella enzyme activity. The rationale behind the oxygen‐to‐carbon substitution in **5** is that the PDE activity in SidE enzymes relies on expelling AMP. Replacing the diphosphate with a methylene bisphosphonate prevents this step from occurring, thereby blocking SidE‐mediated conjugation to substrate proteins.^[8]^ This stabilized Ub^me‐ADPr^ conjugate **5** would thus be able to capture the Legionella enzyme and function as a suitable nonhydrolyzable probe to target such enzymes. Additionally, little is known about the role of the phosphoribosyl residue that remains on the Ub moiety after Dup‐mediated hydrolysis of the targeted substrate protein, and we envision Ub^Pr^‐based tools, such as **6**, to be essential to decipher the role of Ub^Pr^.

## Results and Discussion

The inherent incompatibility of ADPr and other nucleotide‐based structures with strongly acidic conditions routinely used in fluorenylmethoxycarbonyl (Fmoc)/*tert*‐butyloxycarbonyl (Boc) solid‐phase peptide synthesis (SPPS) prohibits the total chemical synthesis of large ADPr peptides or proteins and only allows for the construction of relatively short ADPr peptides by adapting protecting‐group schemes.^[9]^ This has triggered the development of modular synthetic approaches towards such structures,[^7a^, ^10^] in which the polypeptide can be treated with a strong acid to remove protecting groups and be released from a peptide synthesis resin followed by HPLC purification, before it is attached to the delicate ADPr moiety. To allow this final conjugation step to be executed under mild conditions, we envision click chemistry to be the most effective strategy.^[7a]^ Upon substituting Arg42 of Ub with azidohomoalanine through SPPS, conjugation can be achieved at physiological pH with a minimum of chemical additives (3 mm CuSO_4_, 20 mm sodium ascorbate, and 3 mm tris[(1‐benzyl‐4‐triazolyl)methyl]amine (TBTA) ligand) to the α‐oriented propargyl ether on the anomeric position of the riboside in ADPr (**1)**, me‐ADPr (**2)**, or Pr (**3**) (Figure 2 A). The Ub^ADPr^ conjugate formed in such a CuAAC reaction carries a triazole linkage between the ribose and peptide part, from now on referred to as ^triazole^Ub^ADPr^, thus slightly deviating from the native arginine guanidinium linkage (Figure 2 B).

After the successful CuAAC reactions of **1**, **2**, and **3** to Ub carrying an azidohomoalanine mutation on position 42, triazole‐linked ^triazole42^Ub^ADPr^ (**4**), ^triazole42^Ub^me‐ADPr^ (**5**), and ^triazole42^Ub^Pr^ (**6**) were obtained. We set out to compare these triazole‐linked conjugates, and natively linked ^Arg42^Ub^ADPr^, which was prepared enzymatically by using a SdeA mutant, for their affinity towards DupA.^[1b]^

**Figure 3:**
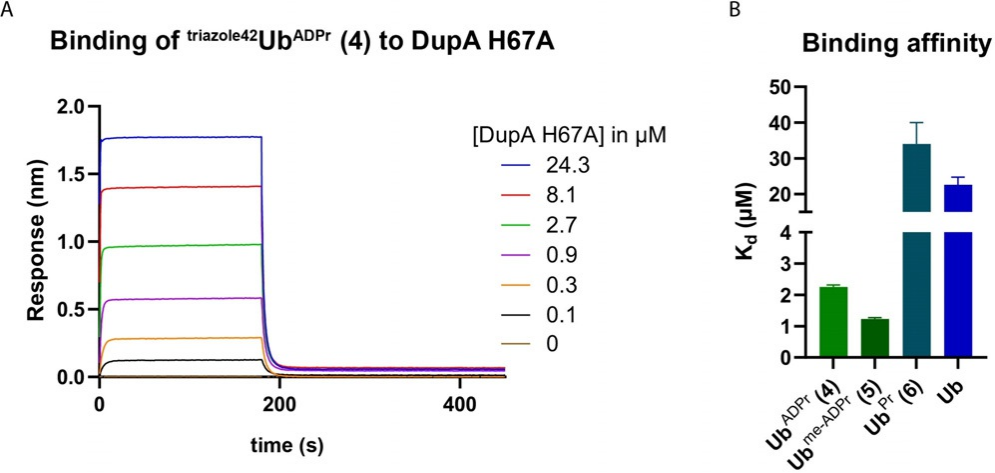
Results of BLI analysis. A) Concentration‐dependent response curves of **4** to DupA‐H67A. B) A comparison of the binding affinities of **4**, **5**, **6**, and Ub.

To this end, we used biolayer interferometry (BLI), and repeated the assay that was described earlier, by immobilizing the different Ub analogues on streptavidin (SA) biosensor tips through the biotin handle attached on the N terminus of Ub, and using glutathione S‐transferase (GST)‐tagged DupA‐H67A as the analyte. With this setup, conjugates **4** and **5** show very high affinities of 11.2 and 10.6 nm, respectively, which are comparable to the *K*_d_ value of 5.7 nm observed for native ^Arg42^Ub^ADPr^ (Figure S3 A in the Supporting Information).^[4a]^ However, the observed nanomolar affinity for unmodified Ub (54.5 nm) would render all DupA inside a human cell bound to unmodified Ub (product‐like) and unavailable for catalysis. We repeated the experiment with the catalytically inactive mutant, DupA‐H67A, lacking the GST tag (Figure 3 A). The results obtained show biologically plausible *K*_d_ values of 2.2 and 1.2 μm for **4** and **5**, respectively, whereas **6** and unmodified Ub have at least a 15‐fold reduced affinity (Figure 3 B and Figure S3 B in the Supporting Information). The discrepancy between the two assays could potentially be attributed to an artefact arising from dimerization of the GST‐tagged analyte that we cannot fully explain at this point (see Figure S3 D in the Supporting Information).

The resulting *K*_d_ values in the absence of the GST tag make biological sense and would fit with the mechanism of the hydrolase, which accepts substrates linked through a phosphodiester bond to ribosylated Ub, with micromolar affinity, and releases the phosphomonoester Ub^Pr^ product due to the lower affinity of the latter.

**Figure 4:**
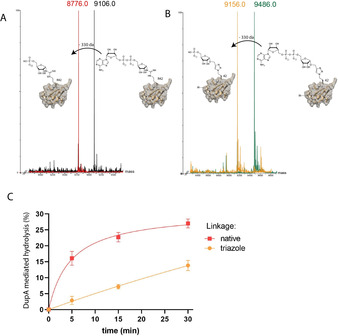
Conversion of Ub^ADPr^ to Ub^Pr^ by DupA followed by mass spectrometry. A) DupA‐mediated hydrolysis of native ^Arg42^Ub^ADPr^. B) DupA‐mediated hydrolysis of **4**, C) Hydrolysis of native ^Arg42^Ub^ADPr^ and **4** by DupA over time.

Next, we wondered whether DupA could hydrolyze **4** to form ^triazole^Ub^Pr^, as reported previously for native ^Arg42^Ub^ADPr^.^[4a]^ We indeed observed robust hydrolysis of natively linked ^Arg42^Ub^ADPr^ (1 μm) by 500 nm DupA after incubation for 1 h at 37 °C (Figure 4 A). Upon applying the same conditions to triazole‐linked **4**, we observed a similar hydrolysis reaction and formation of phosphoribosyl Ub **6**, as monitored by means of mass spectrometry (Figure 4 B), whereas DupA was not able to mediate hydrolysis of stabilized **5** (see Figure S4 in the Supporting Information). In control experiments on both native ^Arg42^Ub^ADPr^ and triazole‐linked **4** in the absence of DupA, only a minor amount of hydrolysis of the pyrophosphate bond is observed, which is most likely due to the acidic conditions employed during mass spectrometry. DupA‐mediated hydrolysis can be attributed to the catalytic specificity of the enzyme because control experiments with triazole‐linked ^triazole54^Ub^ADPr^, ^triazole72^Ub^ADPr^, and ^triazole74^Ub^ADPr^ showed neither hydrolysis nor formation of the corresponding Ub^Pr^s. To investigate this further, we assessed these control compounds for DupA affinity using our BLI setup (Figure S3 C in the Supporting Information). We could not detect significant binding of ^triazole54^Ub^ADPr^ or ^triazole74^Ub^ADPr^ to DupA H67A, giving a clue to why they are not processed by DupA. For ^triazole72^Ub^ADPr^, however, we could detect binding to DupA H67A with a *K*_d_ of 9.3 μm, suggesting that the adenosine moiety could be positioned in a manner resembling the configuration present in **4**, but so that the diphosphate linkage is not oriented appropriately for hydrolysis towards ^triazole72^Ub^Pr^. To investigate any differences in catalysis of DupA on **4** or native ^Arg42^Ub^ADPr^, we followed DupA‐mediated Ub^Pr^ formation over time by mass spectrometry using a lower enzyme concentration of DupA (30 nm) on 3 μm of both hydrolyzable substrates. We observed a clear reduction in velocity (3.5‐fold), when comparing relative *V*_max_ for DupA‐mediated hydrolysis of triazole‐linked **4** to that of native ^Arg42^Ub^ADPr^ (Figure 4 C). It is apparent that, although accepted by DupA, triazole‐linked **4** is hydrolyzed at a reduced rate relative to that of native ^Arg42^Ub^ADPr^. Most likely, this reduced cleavage rate is caused by the more sterically demanding and rigid triazole linkage.

**Figure 5:**
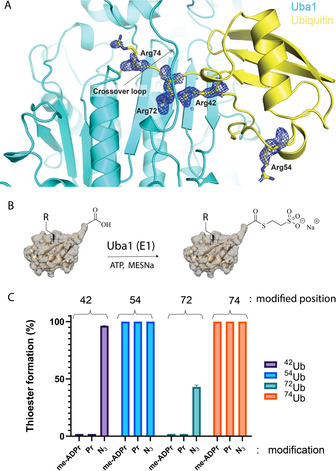
A) Crystal structure of yeast Uba1 in complex with Ub, highlighting the four arginine residues and their corresponding 2 *F*_o_−*F*_c_ electron density map contoured at 2.0 *σ* to illustrate enhanced mobility of Arg54 and Arg74 (PDB ID: 6ZQH). B) Reaction scheme of E1‐mediated thioester formation on the C‐terminal Gly76 of modified Ub by Uba1. C) Relative thioester product formation on ^(triazole42/54/72/72)^Ub^me‐ADPr/Pr/N3^ (10 μm) by human Uba1 (250 nm) after 45 min, as measured by mass spectrometry. An average of three distinct experiments is shown.

ADPribosylation or phosphoribosylation of Arg42 in Ub impairs the conventional ubiquitination machinery because activation by E1, *trans*‐thioesterification to E2, and E3‐mediated discharge from the E2 were shown to be compromised upon the introduction of the modification by Legionella ligase SdeA.^[1b]^ From the crystal structure of ^Arg42^Ub^Pr^, it becomes apparent that any modification of Arg42 or Arg72 will interfere with Ub binding to E1, which could explain the inability of E1 to activate the Ub^Pr^ molecule.^[1b]^ These two arginine residues are reported to be critical in the interaction with the E1 enzyme Uba1, as in a previous study mutations of Arg42 or Arg72 to leucine were shown to result in a dramatically lower affinity between the E1 enzyme and Ub adenylate.^[11]^ In addition, residue 72 is crucial for determining Ub‐like specific recognition by E1, where for Ub this residue is an arginine, for Nedd8 it is an alanine, and for SUMO‐family members it is either a glutamate or glutamine residue.^[12]^ We managed to improve the resolution of our previously reported X‐ray structure of *Saccharomyces cerevisiae* Uba1 in complex with Ub from crystals diffracting anisotropically to 2.03 Å (Figure 5 A), which shows the C‐terminal tail of Ub reaching towards the adenylation site of Uba1. This yeast homolog of Uba1 has a conserved overall structure with high sequence identity (68 %) in the active adenylation domain compared to human Uba1.^[13]^ Figure 5 A shows the crossover loop connecting the adenylation domain to the catalytic cysteine domain encompassing the C‐terminal tail of Ub just above the Arg42 and Arg72 guanidinium groups of Ub. The close spatial positioning of these residues could explain our observation that Ub ADPribosylated at Arg72 can still bind to DupA.

**Figure 6:**
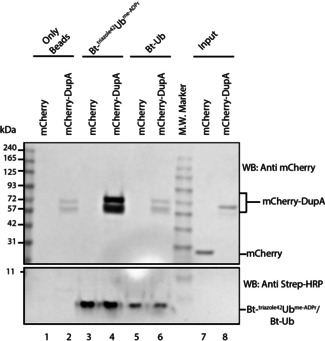
Western blot showing pull‐down with biotin–**5** or Ub on lysate of mCherry‐DupA or mCherry‐only overexpressing HEK293T cells.

Furthermore, we observe a weak electron density for the guanidinium groups of Arg54 and Arg74 in this structure, indicating flexibility and the possibility for these residues to adopt multiple conformations. Both the *σ*‐weighted 2 *F*_o_−*F*_c_ electron density map and the *B* factors of the guanidinium groups of the arginine residues suggest that Arg42 and Arg72 remain in a more rigid conformation, as part of the binding interface with the Uba1 adenylation domain, compared to Arg74 and Arg54. The *B* factors of the CZ atom of the guanidium groups of Arg42, Arg54, Arg72, and Arg74 of Ub are 38.5, 63.7, 30, and 55.9 Å^2^, respectively. The guanidinium groups of Arg42 and Arg72 show well‐defined electron densities, indicative of their fixed placement in a single conformation, necessary for binding to the adenylation domain of Uba1. To validate whether the triazole‐linked Ub^ADPr^ analogues would interfere with Uba1‐mediated activation of Ub, we incubated ^triazole42^Ub^me‐ADPr^ **5**, ^triazole54^Ub^me‐ADPr^, ^triazole72^Ub^me‐ADPr^, ^triazole74^Ub^me‐ADPr^, ^triazole42^Ub^Pr^ **6**, ^triazole54^Ub^Pr^, ^triazole72^Ub^Pr^, and ^triazole74^Ub^Pr^ with human Uba1 (E1) in the presence of sodium 2‐sulfanylethanesulfonate (MESNa) and ATP, and monitored thioester formation using mass spectrometry (Figure 5 B). It became apparent that both me‐ADPr and Pr modification of positions 72 and 42 completely abolished formation of the Ub‐Gly76‐ MESNa thioester, whereas the same modifications at positions 54 and 74 had no effect since efficient thioester formation was observed. When using Arg‐to‐azidohomoalanine Ub mutants, the precursors used for click chemistry, all azido‐containing mutants were accepted and processed by the E1 enzyme to form Ub‐MESNa thioesters (bars labeled N_3_ in Figure 5 C). Notably, the Arg72‐Aha mutant was significantly slower and Arg42‐Aha was moderately slower in thioester formation as only 43(±1.6) % and 96(±0.1) %, respectively, of the thioester was formed in the same time frame that the 54 and 74 mutants needed to reach full conversion. Upon longer incubation, the 72 mutant also reached complete conversion to the MESNa thioester (see Figure S5 in the Supporting Information). It became apparent that changes in the chemical properties of the Arg42 and Arg72 guanidinium groups were tolerated since changing them to azides only slowed down E1‐mediated thioester formation, but did not completely abolish the activity. The introduction of the larger Pr or me‐ADPr modification on either Arg42 or Arg72, however, does lead to a complete loss of thioester formation. This can potentially be explained by the steric bulk of the modification clashing with the E1 crossover loop and/or the negative charge present on the modification, which might play a role in electrostatic repulsion by the negatively charged pocket of E1 that normally accommodates the positively charged Arg72 guanidinium group of Ub.^[12]^ These results again reflected similar behavior of native arginine‐linked ^Arg42^Ub^ADPr^ and the modified Ub analogues, which, although carrying the triazole linkage, show a comparable affinity and biochemical functioning. We were eager to see if these tools could indeed be used as probes. For this purpose, we decided to test whether DupA‐WT could be pulled down from cell lysates using biotinylated **5** as bait. HEK293T cells were transfected with mCherry or mCherry‐DupA‐WT and lysates were prepared to perform a pull‐down experiment under nondenaturing conditions. mCherry is an optimized fluorescent protein tag that allows for visualization of the tagged protein of interest in cells, as well as their pull‐down and visualization in Western blotting with the anti‐mCherry antibody.^[14]^ It is important to bear in mind that, compared with other Ub activity‐based probes, in which a covalent complex is formed upon action of the targeted enzyme on the probe (e.g., a DUB capturing a Ub‐VS, Ub‐VME, or Ub‐Prg probe by means of cysteine catalysis),^[15]^ in our case, the interaction with biotinylated **5** does not lead to a covalent complex and solely relies on its intrinsic affinity. We found that probe **5** was able to bind and enrich DupA‐WT (Figure 6, lane 4), whereas controls with either mCherry (lane 3) or a nonspecific interaction with the SA beads (lane 2) showed no or only minimal DupA recovery, respectively. Similarly, pull‐down with biotin‐Ub only showed marginal enrichment for DupA (Figure 6, lane 6) to a comparable extent to that in the beads‐only control. We repeated this experiment with a slight excess of biotin‐Ub and quantified these results using densitometry, showing >10‐fold enrichment of mCherry‐DupA recovery by biotinylated **5** compared with biotinylated wild‐type Ub or beads (Figure 6). We then performed pull‐downs from HEKT293T cell lysate using nonhydrolyzable probes **5** and ^triazole72^Ub^me‐ADPr^ and subjected the interacting proteins to trypsin digestion and MS/MS analysis to compare their interactome versus native Ub (Figure S7 in the Supporting Information).

Intriguingly, both sites of ADPribosylation lead to increased interaction with distinct proteins compared with that of unmodified Ub, such as the Ub ligase MYCBP for ^triazole72^Ub^me‐ADPr^ and Ub ligase TRIM28 for **5**, as well as the deubiquitinating enzyme OTUD4 for **5**. A decreased interaction with the deubiquitinating enzyme USP5 is observed for both sites of modification in comparison with unmodified Ub. The change of interaction partners for Ub^me‐ADPr^ contains, among others, deubiquitinating enzymes, Ub ligases, and proteins involved in intracellular (endosomal) trafficking or endoplasmic reticulum–Golgi maintenance. These initial results need further validation and are a worthy subject of further research, to define the underlying cellular pathways wherein Ub ADPribosylation plays a role.

## Conclusion

The preparation of ADP‐ribose, adenosine methylenebisphosphonate ribose, and phosphoribose carrying an α‐oriented alkyne on the anomeric position allowed us to conjugate azidohomoalanine‐modified biotin‐Ub through CuAAC cycloadditions on all four arginine positions in Ub (42, 54, 72, and 74). This modular approach ensured the construction of Ub^ADPr^ analogues that were used to study a deubiquitinating enzyme from the Legionella bacterium and a mammalian canonical Ub activating enzyme, the activity of which was shown to be affected by modifications of Ub caused by Legionella infection. We found that the Legionella effector enzyme DupA had a high affinity for chemically prepared triazole‐linked **4**, which was comparable to that of natively linked ^Arg42^Ub^ADPr^, although hydrolysis experiments showed that the rate of cleavage was reduced for triazole‐linked Ub^ADPr^. We furthermore demonstrate that DupA was a site‐specific hydrolase since 54‐, 72‐, and 74‐Ub^ADPr^ were not converted into the corresponding Ub^Pr^s. The thioester‐forming activity at the C‐terminal Gly76 of Ub by Uba1 was fully abrogated if positions 42 or 72 carried me‐ADPr or Pr modifications, but only reduced in speed if azidohomoalanine was introduced at those positions. Neither me‐ADPr nor Pr modifications at positions 54 or 74 had any influence on the E1‐mediated reaction, whereas positions 42 and 72 were found to be critical. These experiments, in combination with detailed insights from the high‐resolution structure, further established that, by modifying Arg42, Legionella was able to block activation of Ub mediated by the canonical ubiquitination cascade. In addition, the affinity of **5** for DupA allowed such tools to be used as a noncovalent probe to enrich overexpressed mCherry‐tagged DupA from HEK293T cell lysates. Because structural homology between bacterial enzymes is often poor and similarity searches have not yet identified any other bacterial or mammalian enzymes involved in the Pr‐ubiquitination pathway, we envision the molecular tools prepared herein to be of great value in answering, in the near future, the question whether this unusual ligase and hydrolase machinery plays a role in other organisms besides Legionella.

## Conflict of interest

The authors declare no conflict of interest.

## Supporting information

As a service to our authors and readers, this journal provides supporting information supplied by the authors. Such materials are peer reviewed and may be re‐organized for online delivery, but are not copy‐edited or typeset. Technical support issues arising from supporting information (other than missing files) should be addressed to the authors.

Supplementary
