# Supplementary material for: Development of ADPribosyl Ubiquitin Analogues to Study Enzymes Involved in Legionella Infection
Source: Chemistry. 2020 Dec 23;27(7):2506–12. doi: 10.1002/chem.202004590 (PMC7898697; doi:10.1002/chem.202004590)
Supplement: Supplementary file 1 — Supplementary [file CHEM-27-2506-s001.pdf]

Chemistry–A European Journal

Supporting Information

Development of ADPribosyl Ubiquitin Analogues to Study Enzymes Involved in Legionella Infection

Robbert Q. Kim,[a] Mohit Misra,[b, c, d] Alexis Gonzalez,[b, c] Ines Tomašković,[b, c] Donghyuk Shin,[b, c, f] Hermann Schindelin,[d] Dmitri V. Filippov,[e] Huib Ovaa†,[a] Ivan Đikić,[b, c] and Gerbrand J. van der Heden van Noort*[a]

Supporting Information

Supporting Information Figure 3 2
Supporting Information Figure 4. 3
Supporting Information Figure 5. 4
Supporting Information Figure 6. 4
Supporting Information Figure 7. 5
Supporting Information table 1. 6
General Procedures 7
Synthesis of propargyl ADPr derivatives..11
Copper catalyzed Huisgen 1,3 -dipolar cycloaddition reactions 13
NMR spectra of compounds 16

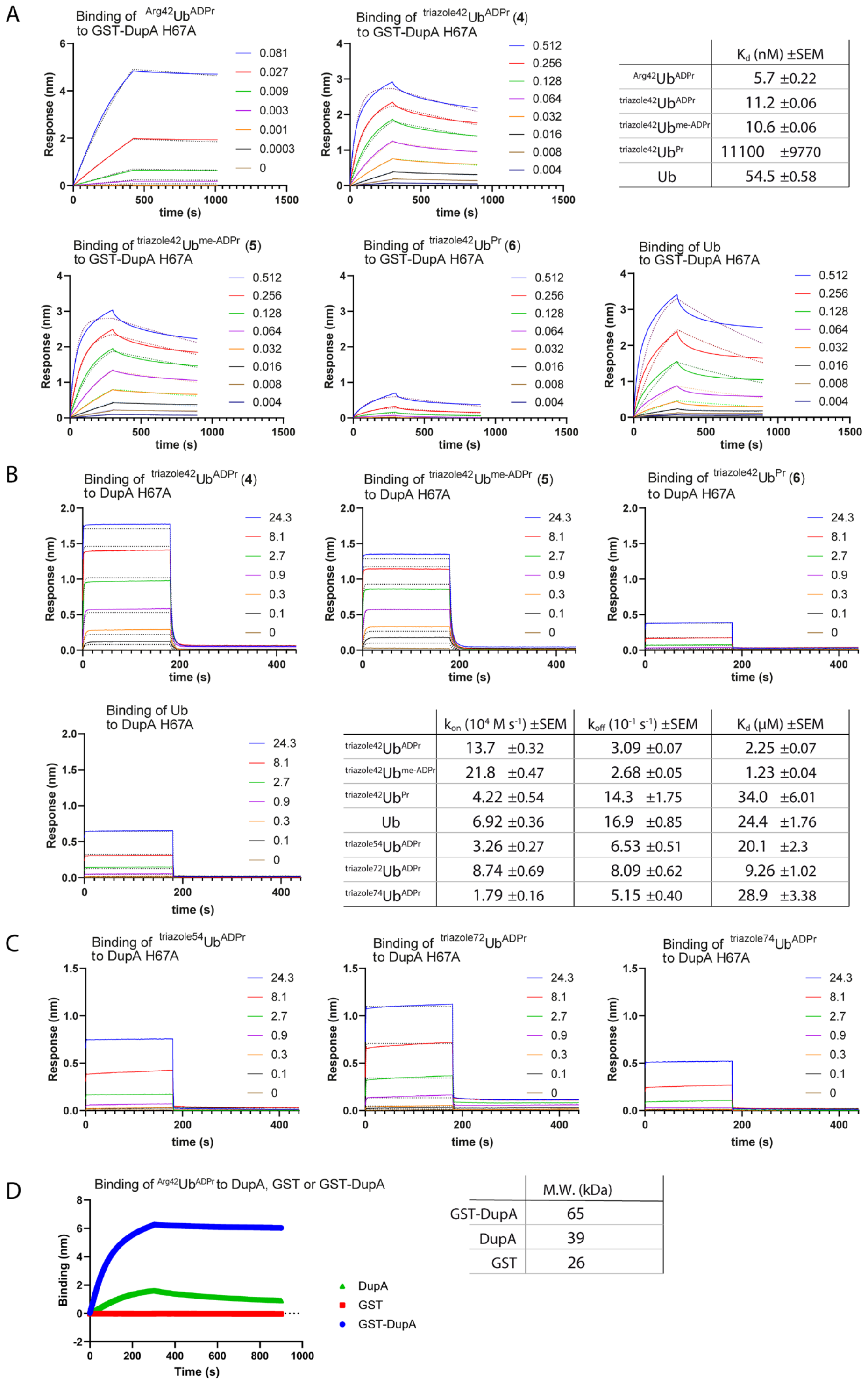

	K_d (nM) ±SEM
$^{Arg42}Ub^{ADPr}$	5.7 ±0.22
$^{triazole42}Ub^{ADPr}$	11.2 ±0.06
$^{triazole42}Ub^{me-ADPr}$	10.6 ±0.06
$^{triazole42}Ub^{Pr}$	11100 ±9770
Ub	54.5 ±0.58

	k_{on} (10^4 M s^{-1}) ±SEM	k_{off} (10^{-1} s^{-1}) ±SEM	K_d (μM) ±SEM
$^{triazole42}Ub^{ADPr}$	13.7 ±0.32	3.09 ±0.07	2.25 ±0.07
$^{triazole42}Ub^{me-ADPr}$	21.8 ±0.47	2.68 ±0.05	1.23 ±0.04
$^{triazole42}Ub^{Pr}$	4.22 ±0.54	14.3 ±1.75	34.0 ±6.01
Ub	6.92 ±0.36	16.9 ±0.85	24.4 ±1.76
$^{triazole54}Ub^{ADPr}$	3.26 ±0.27	6.53 ±0.51	20.1 ±2.3
$^{triazole72}Ub^{ADPr}$	8.74 ±0.69	8.09 ±0.62	9.26 ±1.02
$^{triazole74}Ub^{ADPr}$	1.79 ±0.16	5.15 ±0.40	28.9 ±3.38

	M.W. (kDa)
GST-DupA	65
DupA	39
GST	26

Supporting Information Figure 3.

Bio Layer Interferometry analyses of various binding experiments, all graphs show concentrations in μM and fitted curves as dotted lines. A) Binding experiments were performed by immobilizing either natively-linked $^{Arg42}Ub^{ADPr}$, $^{triazole42}Ub^{ADPr}$ (**4**), $^{triazole42}Ub^{me-ADPr}$ (**5**), $^{triazole42}Ub^{Pr}$ (**6**) or Ub and subsequently assessing their binding to the mentioned concentrations of GST-tagged DupA-H67A [1]. The data were fit using the Octet96 software, leading to biologically unlikely K_d-values (table: K_d-values in

nM). The lack of any plateau in the association phase already indicates some issues in the experimental set up, possibly an artefact introduced by the GST-tag, as further discussed in *D*. B) The same experiments as in *A* were carried out using DupA-H67A. On- and off-rates were fitted using the Octet96 software, with the resulting values depicted in the table. C) Binding data of the ADPr modification on different arginines of ubiquitin indicate a huge reduction in affinity, compared to $^{triazole42}Ub^{ADPr}$ shown in *B*. For comparison, $k_{on/off}$-rates are noted in the table in *B*. D) Comparison of BLI-measurements of the interaction of native $^{Arg42}Ub^{ADPr}$ and GST-DupA H67A, DupA H67A and GST only. The amplitude of the signal, which is proportional to the molecular weight increase, was larger (~2-fold) than expected on the basis of the molecular weight of the GST-DupA:$^{Arg42}Ub^{ADPr}$ complex. Our hypothesis is that the GST-tag is responsible for GST-DupA dimerization on the chip. This additional interaction interferes with determining the proper K_D's. The GST-tag itself did not directly bind to $^{Arg42}Ub^{ADPr}$.

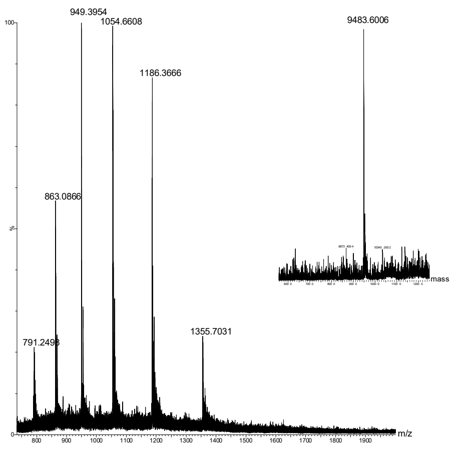

42 Ub^{AMPr} 0 min

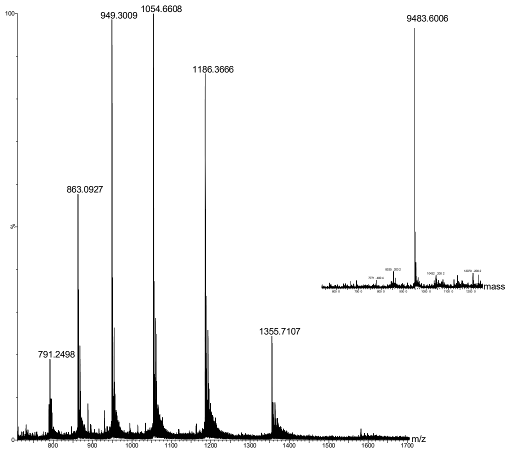

42 Ub^{MPr} 45 min

Supporting Information Figure 4.

Mass spectra of DupA proteolysis on $^{triazole42}Ub^{me\text{-}ADPr}$ at 0 min and after 45 min incubation at 37 °C.

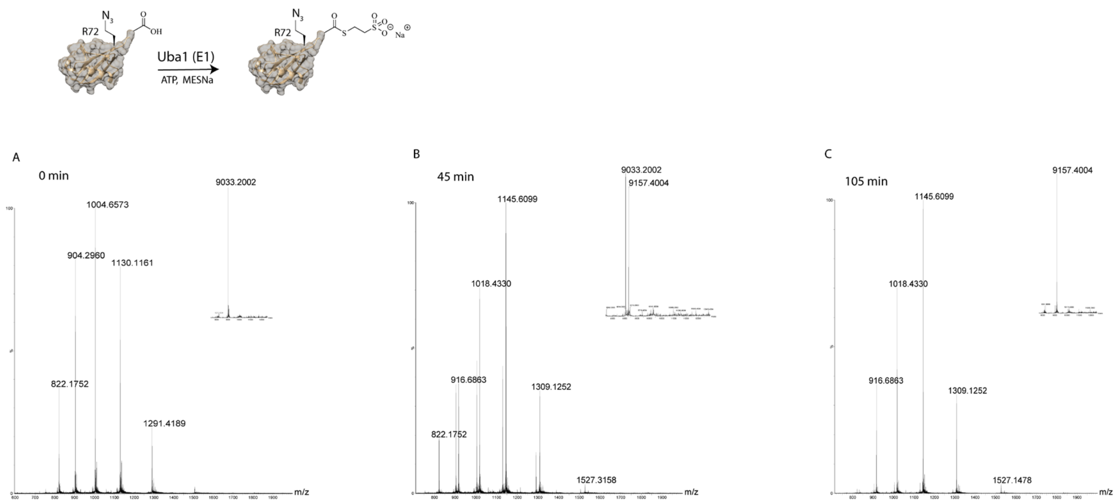

Supporting Information Figure 5.

E1-mediated thioester formation on ^{Aha72}Ub after A) 0 min, B) 45 min and C) 105 min incubation at 37 °C.

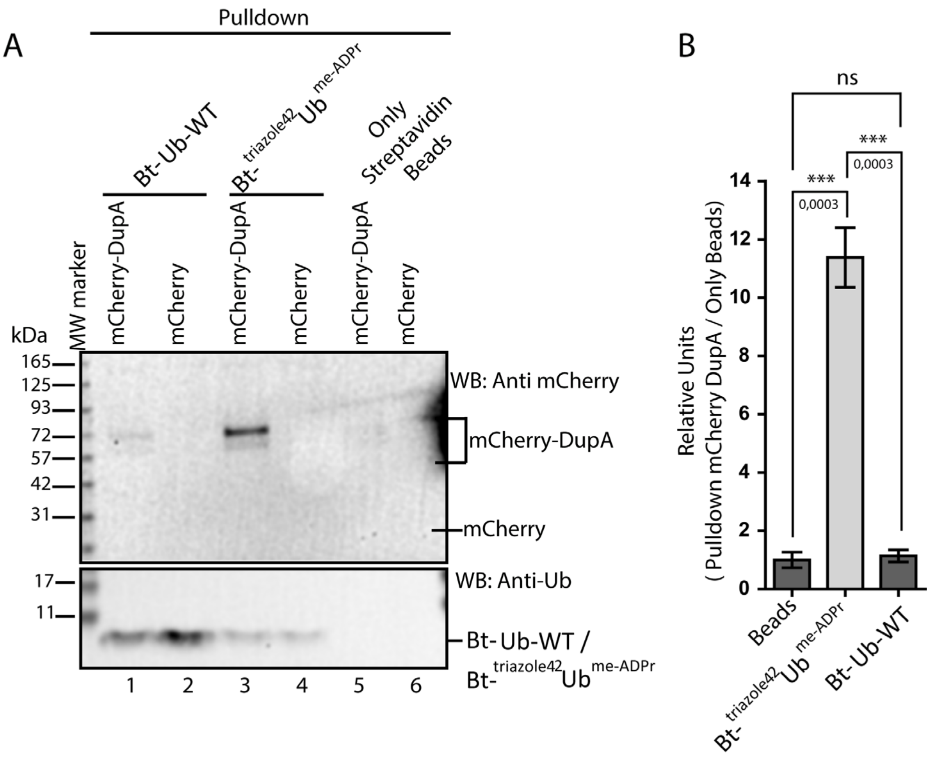

Supporting Information Figure 6.

Western-blot showing pull-down using biotin-^{triazole42}Ub$^{me\text{-}ADPr}$ (**5**) or Ub on lysate of mCherry-DupA or mCherry-only over-expressing HEK293T cells, B) densiometric analysis of western-blot from *A)* showing relative enrichment in mCherry-DupA signal vs beads only signal using biotin-^{triazole42}Ub$^{me\text{-}ADPr}$ (**5**) or Ub.

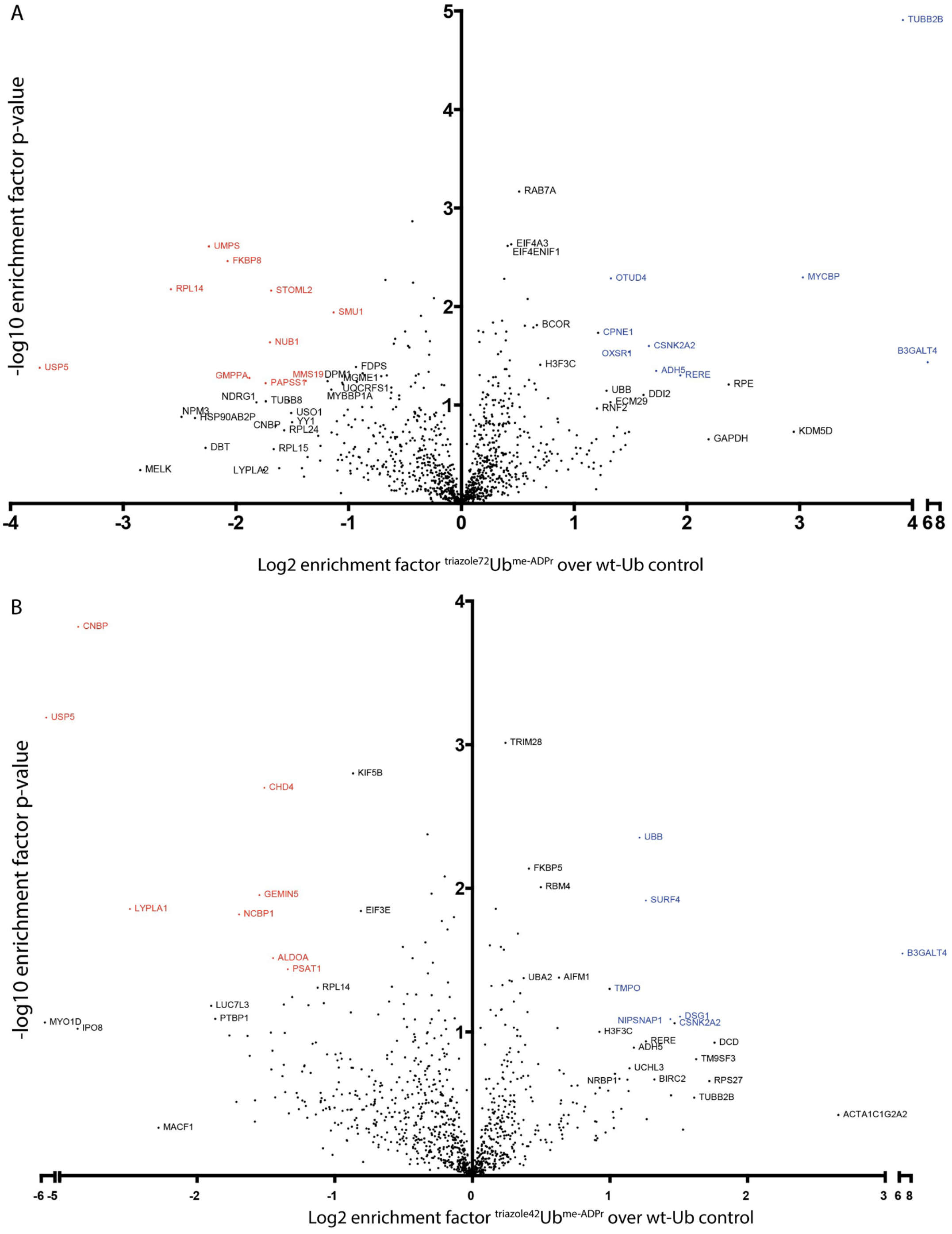

Supporting Information Figure 7.

Volcano plots of proteomic analysis of pulldown from HEK293T cell lysate using A) Bt-$^{triazole72}Ub^{me\text{-}ADPr}$ versus Bt-Ub control, B) Bt-$^{triazole42}Ub^{me\text{-}ADPr}$ versus Bt-Ub control.

Supporting Information table 1.

Crystallographic data collection and refinement statistics

Uba1Ub (PDB code: 6ZQH)	
Wavelength (Å)	0.9184
Beamline	BESSY 14.1
Resolution range (Å)	46.16 - 2.026 (2.105 - 2.026)
Space group	P 2 21 21
Unit cell (Å, °)	76.87 173.17 214.81 90 90 90
Total reflections	735705 (35132)
Unique reflections	131321 (6564)
Multiplicity	5.6 (5.4)
Completeness Spherical (%)	70.25 (4.26)
Completeness Ellipsoidal (%)	94.81 (65.56)
$\langle I/\sigma(I)\rangle$	6.48 (1.09)
Wilson B-factor (Å^2)	27.4
R-merge	0.242 (1.639)
R-meas	0.266 (1812)
R-pim	0.109 (0.764)
$CC_{1/2}$	0.9896 (0.3568)
R-work	0.1951 (0.2972)
R-free	0.2271 (0.3234)
Number of non-hydrogen atoms	18,867
macromolecules	17,253
ligands	53
solvent	1,561
RMS deviations in bond lengths (Å)	0.003
RMSdeviations in bond angles (°)	0.65
Ramachandran favored (%)	97.35
Ramachandran allowed (%)	2.56
Ramachandran outliers (%)	0.09
Rotamer outliers (%)	1.65
Clashscore	2.98
Average B-factors (Å^2)	39.02
macromolecules	39.16
ligands	47.95
solvent	37.13

Statistics for the highest-resolution shell are shown in parentheses.

[a]R_{sym}= $\Sigma_{hkl}\Sigma_i |I_i - \langle I\rangle| / \Sigma_{hkl}\Sigma_i I_i$ where I_i is the ith measurement and $\langle I\rangle$ is the weighted mean of all measurements of *I*. [b]$R_{pim} = \Sigma_{hkl} 1/(N-1)^{1/2} \Sigma_i |I_i - \langle I\rangle| / \Sigma_{hkl}\Sigma_i I_i$, where N is the redundancy of the data and $I(hkl)$ the average intensity. [c]$\langle I/\sigma I\rangle$ indicates the average of the intensity divided by its standard deviation. [d]$R = \Sigma_{hkl} ||F_o| - |F_c|| / \Sigma_{hkl}|F_o|$ where F_o and F_c are the observed and calculated structure factor amplitudes. [e]R_{free} same as R for 5% of the data randomly omitted from the refinement. The number of reflections includes the R_{free} subset.

General Procedures

General reagents were obtained from Sigma Aldrich, Fluka and Acros and used as received. Solvents were purchased from BIOSOLVE or Aldrich. Peptide synthesis reagents were purchased from Novabiochem. Column chromatography was carried out on silica gel (0.035-0.070 mm, 90 Å, Acros). Nuclear magnetic resonance spectra (^{1}H-NMR, ^{13}C-NMR, ^{31}P-NMR) were determined using a Bruker AV-500 or AV-600 (500/600 MHz) at 298 K. Multiplicities in NMR spectra are indicated with the symbols 'd' (doublet), 'dd' (double doublet), 's' (singlet), 't' (triplet) and 'm' (multiplet). Chemical shifts are given in ppm (δ) relative to TMS (0 ppm) or indirectly referenced to H_3PO_4 (0.00 ppm) in D_2O via the solvent residual signal.

LC-MS measurements

Waters 2795 Separation Module (Alliance HT) using a Phenomenex Kinetex C18-column (2.1x50, 2.6 µm), Waters 2996 Photodiode Array Detector (190-750 nm) and LCT™ ESI-Mass Spectrometer. Samples were run using 2 mobile phases: A = 1% CH_3CN, 0.1% formic acid in water and B = 1% water and 0.1% formic acid in CH_3CN. Flow rate= 0.8 mL/min, runtime= 6 min, column T= 40°C. Gradient: 0 - 95% B. Data processing was performed using Waters MassLynx Mass Spectrometry Software 4.1 (deconvolution with MaxEnt1 function).

RP-HPLC purifications

A) Waters preparative RP-HPLC system, equipped with a Waters C18-Xbridge 5 µm OBD (30 x 150 mm) column at a flowrate of 37.5 mL/min using 3 mobile phases: A: MQ, B: CH_3CN and C: 1% TFA in MQ. Gradient: 20 -> 45% B, 5% C.
B) Shimadzu semi-preparative RP-HPLC system, equipped with a Waters C18-Xbridge 5 µm OBD (10 x 150 mm) column at a flowrate of 6.5 mL/min. using 2 mobile phases: A: MQ + 0.05% FA, B: CH_3CN + 0.05 % FA. Gradient: 0 -> 15% B.

Anion Exchange Chromatography

Anion Exchange Chromatography was performed on a Resource Q (6 mL) column (GE Healthcare) using a gradient from 10 mM NH_4OAc to 1 mM NH_4OAc in 5 column volumes at pH 8.

Size Exclusion Chromatography

Size Exclusion Chromatography for ADPr-Ub proteins was performed on a Superdex SD75 16/600 column (GE Healthcare), using a 20 mM TRIS, 150 mM NaCl buffer at pH 7.6.

HRMS-measurements

High resolution mass spectra were recorded on a Waters XEVO-G2 XS Q-TOF mass spectrometer equipped with an electrospray ion source in positive mode (source voltage 3.0 kV, desolvation gas flow 900 L/hr, temperature 250 °C) with resolution R = 22000 (mass range m/z = 50-2000) and 200 pg/µL Leu-Enk (m/z = 556.2771) as a "lock mass".

Solid Phase Peptide Synthesis

SPPS was performed on a Syro II MultiSyntech Automated Peptide synthesizer using standard 9-fluorenylmethoxycarbonyl (Fmoc) based solid phase peptide chemistry on a 25 µmol scale, using a fourfold excess of amino acids relative to pre-loaded Fmoc amino acid Wang type resin (0.2 mmol/g, Applied Biosystems®) or pre-loaded Fmoc amino acid trityl resin (0.2 mmol/g, Rapp Polymere GmbH). The Ub (mutant) peptide sequences were synthesized on resin following the procedures described earlier, containing azido homo-alanine at the indicated positions.[2]
Peptides were treated with TFA/TIS/H_2O/Phenol for 2.5 hours followed by precipitation from Et_2O/Pentane. The crude peptide was subsequently purified using RP-HPLC and pure fractions (as monitored by LC-MS) were pooled and lyophilized.

Constructs and Protein Purification

DupA WT and H67A constructs and purification were described previously.[1] The GST-tagged expression constructs were transformed into T7 Express cells and grown in LB with 100 µg mL^{-1} ampicillin at 37°C until an OD_{600} of 0.7. Induction was started using 0.5 mM IPTG and cells were harvested after 5 hours at 20°C.
Cell pellets were resuspended in GST-buffer (50 mM Tris pH 7.5, 150 mM NaCl and 1 mM TCEP) and lysed through sonication (12 cycles, 15s on, 45s off) on ice. Cell debris was spun down with 21,000 G at 4°C and the supernatant was applied to GST-beads equilibrated in GST buffer. Beads were washed extensively with said buffer before the protein was eluted using elution buffer (50 mM Tris pH 7.5, 50 mM NaCl, 25 mM GSH, 1 mM TCEP). Elution fractions were pooled and cleaved overnight at 4℃ with TEV protease to remove the GST tag. The protein solution was concentrated before being applied to S75 16/60 gel filtration in GF buffer (50 mM Tris pH 7.5, 50 mM NaCl, 1 mM TCEP). Fractions containing DupA were pooled, concentrated to ~10 mg mL^{-1} and flash frozen in liquid N2.
For the expression of N-terminally His6-tagged Uba1 from *S. cerevisiae* the previously described pET28a-Uba1 construct encoding for residues 10-1024 was used. The coding sequence for *S. cerevisiae* ubiquitin was introduced into pET30a enabling expression without any fusion tag. Ubiquitin was expressed in *E. coli* BL21(DE3) by induction with 0.1 mM IPTG at an OD_{600} of 0.6-1 followed by overnight growth at 16°C. For expression of yeast His6-TEV-Uba1 *E. coli* BL21(DE3)-RIL cells were induced with 0.1 mM IPTG in TB media (containing 0.5% glucose) at an OD_{600} of 1 followed by overnight growth at 25°C.
Uba1 was purified via a three-step purification starting with Ni-NTA chromatography, followed by hydrophobic interaction chromatography and size exclusion chromatography. To purify tag-free ubiquitin, the acid precipitation method was followed by purification with cation exchange chromatography and size exclusion chromatography.
The human N-terminally His6-tagged Uba1 was expressed and purified as reported by Mulder and coworkers.[3]

Crystallization, Data Collection and Refinement

For crystallization, Uba1 and Ub were mixed in a 1:1 molar ratio and incubated at RT for 30 mins. The crystals were obtained using the liquid handling robot Honeybee 963

in sitting drop vapor diffusion experiments with drops containing 0.5 µl of protein solution mixed with 0.5 µl of mother liquor (0.2 M ammonium formate and 20% PEG 3350) and a reservoir of 40 µl mother liquor in a 96-well crystallization plate sealed with adhesive sealing film with the help of a Roboseal unit and kept at 273 K. The crystals appeared in 5 days and were cryoprotected by soaking in mother liquor supplemented with 15% glycerol before flash-cooling in liquid nitrogen. X-ray diffraction data were collected at BESSY 14.1 [4] beamline (HZB, Berlin) at 100 K. Data were indexed and integrated using XDS.[5] Initial analysis of the data suggested anisotropic diffraction. Reprocessing the data with the Staraniso server (Tickle, I.J., Flensburg, C., Keller, P., Paciorek, W., Sharff, A., Vonrhein, C., Bricogne, G. (2018). STARANISO (http://staraniso.globalphasing.org/cgi-bin/staraniso.cgi). Cambridge, United Kingdom: Global Phasing Ltd.) gave resolution limits of 2.03 Å in the best and 2.86 Å in the worst direction as defined by a CC(1/2) value of greater than 0.3 (Karplus and Diederichs, Science 2012, 25:1030-1033 and Karplus and Diederichs, Curr. Op. Struct. Biol. 2015, 34:60-68) in the highest resolution shell. The phasing of the structure was done using molecular replacement with Phaser using PDB entry 3CMM as initial model. The final refinement was carried out with Phenix [6] resulting in R-values of 0.195/0.227. The structure has been deposited in the PDB (rcsb.org) under ID 6ZQH. Figure 5A was prepared using Pymol (The PyMOL Molecular Graphics System, Version 2.09 Schrödinger, LLC).

Bio Layer Interferometry-measurements

BLI measurements were performed on an OctetRed system (ForteBio). Bt-Ub^{ADPr} linked via triazole42 (**4**), triazole54, triazole72 or triazole74, Bt-Ub$^{me\text{-}ADPr}$ (**5**), Bt-Ub^{Pr} (**6**) or Bt-Ub were loaded on streptavidin-biosensors and equilibrated in buffer (20 mM HEPES, 150 mM NaCl, 0.05% Tween-20, 1 mg/mL BSA, pH 7.4). Subsequently, the sensors were transferred into solutions containing varying concentrations of DupA H67A (0.1 – 24.3 µM) to measure the association of the analyte. Dissociation of the modified Ub:DupA complexes was measured by placing the sensor into buffer again. Dissociation constants (K_d) were calculated using the ForteBio Data Analysis software by co-fitting all concentrations simultaneously.

DupA proteolysis of Ub^{ADPr}

Bt-^{triazole}Ub^{ADPr} (**4**) or native Bt-^{Arg}Ub^{ADPr} (3 µM) in buffer (20 mM TRIS, 150 mM NaCl, 1 mM TCEP, pH 7.6) was incubated with DupA (30 nM) at 37 ^{o}C. At indicated time points (0, 5, 15 or 30 minutes) 5 µL reaction mixture was diluted 10 times and HRMS was measured. The ratio of product versus starting material was determined and corrected for t=0 min and plotted as the increase in product formation over time. The means of three individual measurements is depicted with standard deviation.

E1-mediated thioesterification of modified Ub

Bt-^{triazole}Ub$^{me\text{-}ADPr}$ (**5**), Bt-^{triazole}Ub^{Pr} (**6**), Bt-Ub^{N3} on position 42, 54, 72 or 74 or WT-Ub as positive controle (10 µM) in buffer (125 mM HEPES, 100 mM MESNa, 10 mM $MgCl_2$, 10 mM ATP, pH 7.8) were incubated with human Uba1 (250 nM) at 37 ^{o}C for 45 min. 5 µL reaction mixture was diluted 10 times and HRMS was measured. The ratio of

product versus starting material was determined and plotted as the amount of thioester product formed at the indicated time.

Pulldown of overexpressed mCherry-DupA

HEK293T cells were transfected with either mCherry-DupA (WT version) or mCherry as control and cultured in 10 cm plates. The cells were collected after 16 hours and lysed using lysis buffer (50 mM Tris-HCl pH 7.4, 150 mM NaCl, 1% Triton X-100, protease inhibitor cocktail). Lysates were incubated with biotinylated Ub^{AMPr} (**5**) or biotinylated Ub for 4 hours and subsequently mixed with streptavidin conjugated agarose beads for 1 additional hour at 4 °C. Afterwards extensive washing steps were performed. Proteins were eluted by boiling with 2X gel loading dye (120 mM Tris-HCl pH 6.8, 4% SDS, 20% glycerol), followed by SDS-PAGE on Tris-glycine gel. Western blotting was performed using anti-mCherry and anti-Strep-HRP or anti-Ub antibodies and overlayed with the fluorescent scan to show the molecular weight marker.

Proteomics

HEK293T cells were cultured in 10 cm plates. The cells were collected after 16 hours and lysed using lysis buffer (50 mM Tris-HCl pH 7.4, 150 mM NaCl, 1% Triton X-100, protease inhibitor cocktail). Lysates were incubated with biotinylated ${}^{triazole42}Ub^{me\text{-}ADPr}$, ${}^{triazole72}Ub^{me\text{-}ADPr}$ or biotinylated Ub for 16 hours and subsequently mixed with streptavidin conjugated agarose beads for 1 additional hour at 4 ^{o}C. Afterwards extensive washing steps were performed using lysis buffer (3-5 times). Proteins were digested on-bead using trypsin for 16 hours at 37 ^{o}C. The tryptic peptides were stage tipped and eluted for MS/MS identification. The experiment was performed in duplicate for ${}^{triazole42}Ub^{me\text{-}ADPr}$ and triplicate for ${}^{triazole72}Ub^{me\text{-}ADPr}$ and biotinylated Ub. Graphs depicting the relative difference between $Ub^{me\text{-}ADPr}$ and Ub are given at a p-value of 5%.

Synthesis of propargyl ADPr derivatives

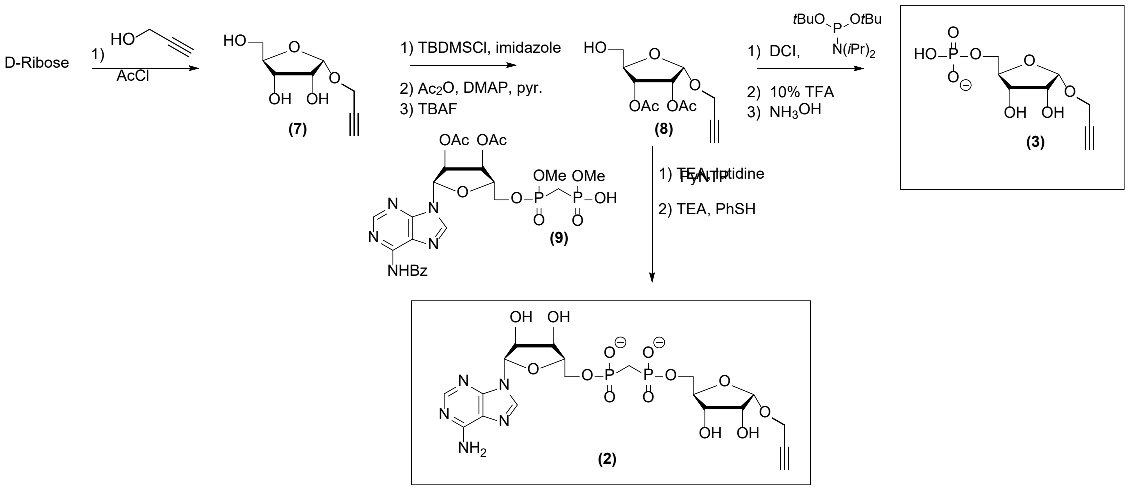

1-O-Propargyl-ribofuranose (7)

D-Ribose was dissolved in propargyl alcohol (5 mL/g) and acetyl chloride (100 µL/g) was slowly added. After stirring at room temperature for 1 hour TLC-analysis (EtOAc) showed complete consumption of the starting material and formation of two new spots (Rf = 0.4, 0.6). The reaction was quenched by adding pyridine (150 µL/g) and concentrated *in vacuo*, followed by co-evaporation with toluene. Silica gel column chromatography resulted in separation of the α and β-anomers. Spectral data is in agreement with that reported by Pfaffe et al.[7]

α- 1-O-Propargyl-ribofuranose (**7**). (1.30 g, 7.0 mmol, 21 %). Rf (EtOAc) = 0.4.
^{1}H NMR (300 MHz, D_2O) δ: 5.25 (d, *J* = 4.4 Hz, 1H, H1'), 4.31 (d, *J* = 1.5 Hz, 2H, CH_2 Prg), 4.14 – 4.07 (m, 2H, H2',H4'), 4.01 (dd, *J* = 6.5, 3.3 Hz, 1H, H3'), 3.68 (AB, J = 13.6, 4.8 Hz, 1H, H5'), 3.60 (AB, J = 14.8, 4.8 Hz, 1H, H5'). **^{13}C NMR** (75 MHz, D_2O) δ: 100.70 (C1'), 85.00 (C4'), 71.23 (C2'), 69.53 (C3'), 61.48 (C5'), 54.95 (CH_2 Prg). **HRMS**: [$C_5H_{12}O_8$ + Na]$^+$: 211.0597 found, 211.0582 calculated.

β -1-O-Propargyl-ribofuranose. (1.50 g, 8.1 mmol, 24 %). Rf (EtOAc) = 0.6.
^{1}H NMR (300 MHz, D_2O) δ: 5.11 (d, *J* = 1.0 Hz, 1H, H1'), 4.34 (q, J = 7.6, 3.2 Hz, 2H, CH_2 Prg), 4.16 (dd, *J* = 7.1, 4.6 Hz, 1H, H2'), 4.04 (dd, *J* = 4.7, 1.0 Hz, 1H, H3'), 4.00 (td, *J* = 6.7, 3.3 Hz, 1H, H4'), 3.78 (dd, *J* = 12.3, 3.3 Hz, 1H, H5'), 3.60 (dd, *J* = 12.4, 6.4 Hz, 1H, H5'). **^{13}C NMR** (75 MHz, D_2O) δ: 105.49 (C1'), 83.07 (C4'), 74.87 (CH Prg), 74.37 (C2'), 70.72 (C3'), 62.67 (C5'), 54.76 (CH_2 Prg). **HRMS**: [$C_5H_{12}O_8$ + Na]$^+$: 211.0597 found, 211.0582 calculated.

α- 1-O-Propargyl-2, 3-di-O-acetyl-ribofuranose (8)

Ribose (**7**) was dissolved in DCM (15 mL/g) and treated with TBDMSCl (1.05 eq) and imidazole (1.50 eq). After stirring at room temperature for 30 min TLC-analysis (EtOAc:Heptane, 1:1 v/v) showed complete consumption of the starting material and formation of a new spot (Rf = 0.55). The reaction mixture was filtered and washed with 1M $KHSO_4$ and 1M NaCl. The organic layers were dried with $MgSO_4$ and concentrated. The crude was dissolved in pyridine (10 mL/g) and treated with Ac_2O (3 mL/g) and catalytic DMAP for 16 hours at room temperature. TLC-analysis (EtOAc:Heptane, 1:1 v/v) showed complete consumption of the starting material and

formation of a new spot (Rf = 0.75). The reaction mixture was concentrated *in vacuo* and coevaporated with toluene twice. The crude was dissolved in THF (20 mL/g) and treated with 0.1 M TBAF. After stirring at room temperature for 30 min TLC-analysis (EtOAc:Heptane, 2:1 v/v) showed complete consumption of the starting material and formation of a new spot (Rf = 0.15). The reaction mixture was diluted in DCM and washed with 1M $KHSO_4$. The organic layers were dried with $MgSO_4$ and concentrated. Silica gel column chromatography afforded the title compound (350 mg, 49%). Spectral data is in agreement with that reported by Liu et al.[8]

^{1}H NMR (300 MHz, D_2O) δ: 5.38 (d, J = 4.5 Hz, 1H, H1'), 5.14 (dd, J = 7.3, 3.5 Hz, 1H, H2'), 4.92 (dd, J = 7.4, 4.5 Hz, 1H. H3'), 4.23 (dd, J = 2.4, 0.7 Hz, 2H, CH_2 Prg), 4.06 (q, *J* = 3.3 Hz, 1H, C4'), 3.70 (t, *J* = 3.1 Hz, 2H, C5'), 2.93 (bs, 1H, OH), 2.41 (t, *J* = 2.4 Hz, 1H, CH Prg), 2.02 (s, 6H, 2x CH_3 OAc). **^{13}C NMR** (75 MHz, D_2O) δ: 170.81, 169.94 (CO OAc), 98.53 (C1'), 82.97 (C4'), 78.78 (Cq Prg), 74.97 (*C*H Prg), 71.06 (C2'), 69.95 (C3'), 62.07 (C5'), 54.53 (CH_2 Prg), 20.85, 20.54 (CH_3 OAc). **HRMS**: $[C_{12}H_{16}O_7 + Na]^+$: 295.0785 found, 295.0781 calculated.

α-propargyl-ADPr (1)

Alcohol (**8**) was phosphorylated *in-situ* and used in pyrophosphate formation as described in detail by Liu et al. [8].

α-propargyl-me-ADPr (2)

Alcohol (**8**) was phosphorylated with protected me-ADP as described by Engelsma et al.[9]. The fully protected methylene bisphosphonate was deprotected using MeCN:TEA:PhSH (3:3:2, 1.6 mL) and stirred for 2 hours at 35 °C. Reaction progression was monitored by LCMS, which indicated complete demethylation after 2 hours. The solution was reduced in vacuo to remove MeCN and pyridine. The residue was dissolved in ammonia (30%, 3 mL) and stirred overnight after which LCMS analysis revealed formation of the product. The excess ammonia was removed by stirring under vacuum. The resulting solution was diluted in water and washed with DCM (3x) to remove excess thiophenol. The water layer was lyophilized to acquire the crude product. Purification by anion exchange chromatography afforded the title compound (2.8 mg, 19%).

^{1}H NMR (300 MHz, D_2O) δ 8.48 (s, 1H, H2-Ade), 8.18 (s, 1H, H8-Ade), 6.03 (d, *J* = 5.6 Hz, 1H, H1"), 5.03 (d, *J* = 3.9 Hz, 1H, H1'), 4.44 (dd, *J* = 5.2, 3.9 Hz, 1H), 4.27 (d, *J* = 3.5 Hz, 1H), 4.15 (dd, *J* = 3.5, 2.4 Hz, 2H, CH_2 Prg), 4.11 – 3.97 (m, 5H), 3.92 – 3.74 (m, 2H), 2.70 (t, *J* = 2.4 Hz, 1H, CH Prg), 2.18 – 2.00 (ap. t, 2H, P-CH_2-P). **^{31}P NMR** (121 MHz, D_2O) δ 17.31, 17.27, 17.14, 17.09. **HRMS**: $[C_{19}H_{27}N_5O_{13}P_2 + H]^+$: found 596.1159, calc. 596.1168

α-propargyl-Pr (3)

Alcohol (**8**) was phosphorylated as described by Liu et al. [8] and isolated using RP-HPLC purification followed by anion exchange chromatography to afforded the title compound (17.6 mg , 21%).
^{1}**H NMR** (300 MHz, D_2O) δ 5.21 (d, *J* = 4.4 Hz, 1H, H1'), 4.25 (dd, *J* = 2.4, 1.1 Hz, 2H, CH_2 Prg), 4.15 (dt, *J* = 3.7, 1.8 Hz, 1H, H4'), 4.11 (dd, *J* = 6.4, 4.4 Hz, 1H, H2'), 4.05 (dd, *J* = 6.3, 2.9 Hz, 1H, H3'), 3.86 (dd, *J* = 5.5, 3.7 Hz, 2H, H5'), 2.77 (t, *J* = 2.4 Hz, 1H, CH Prg). ^{13}**C NMR** (75 MHz, D_2O) δ 100.72 (C1'), 83.86, 83.75 (C4'), 75.73 (CH Prg), 70.99 (C2'), 69.54 (C3'), 64.74, 64.67 (C5'), 54.98 (CH_2 Prg). ^{31}**P NMR** (121 MHz, D_2O) δ 0.40. **HRMS**: $[C_8H_{13}O_8P + H]^+$: 269.0426 found, calc 269.0428.

Copper catalyzed Huisgen 1,3 -dipolar cycloaddition reactions

Biotin-PEG2-^{triazole42}Ub^{ADPr} - Compound (4)

Biotin-PEG2-(R42-Aha)Ub_{76} (5.1 mg), 1.0 eq. was dissolved in 45 µL DMSO and subsequently added to 510 µL buffer (20 mM TRIS/150 mM NaCl, pH 7.6). To this solution 45 µL of freshly prepared click-mixture (1:1:1 v/v/v, $CuSO_4$ (26 mg/mL in water): sodium ascorbate (120 mg/mL in water): TBTA ligand (52 mg/mL in CH_3CN)) was added and the pH was adjusted to 7.4 before addition of 60 µL ADPr-propargyl **1** 2.4 eq. (13 mg/mL in DMSO). The reaction was shaken for 90 min at room temperature and followed using LC-MS analysis. Once the azide starting material was fully converted to the ADPr-conjugate the reaction was quenched using 10 µL EDTA (0.5 M). Dialysis using a 3.5-5 kD MWCO dialysis device removed traces of excess ADPr-propargyl reagent as well as copper/ligand/ascorbate from the reaction, followed by size exclusion chromatography resulting in the title compound (4.6 mg, 0.48 µmol, 84%. Calculated based on SDS-page Coomassie stain comparison with standard curve of WT Ub) . LC-MS (A): Rt = 2.00 min., ESI MS+ (amu) calcd: 9485.36, found 9486.00. HRMS: $[C_{411}H_{675}N_{113}O_{137}P_2S + 7H]^{7+}$: found 1355.9868, calc. 1355.9886, $[C_{411}H_{675}N_{113}O_{137}P_2S + 8H]^{8+}$ found 1186.6187, calc. 1186.6161, $[C_{411}H_{675}N_{113}O_{137}P_2S + 9H]^{9+}$: found 1054.8846, calc. 1054.8818, $[C_{411}H_{675}N_{113}O_{137}P_2S + 10H]^{10+}$: found 949.4930, calc. 949.4944, $[C_{411}H_{675}N_{113}O_{137}P_2S + 11H]^{11+}$: found 863.2679, calc. 863.2684, $[C_{411}H_{675}N_{113}O_{137}P_2S + 12H]^{12+}$: found 791.4152, calc. 791.4133, $[C_{411}H_{675}N_{113}O_{137}P_2S + 13H]^{13+}$: found 730.6146, calc. 730.6129.

Biotin-PEG2-^{triazole42}Ub$^{me-ADPr}$ - Compound (5)

A similar protocol as for compound **4** was used, where Biotin-PEG2-(R42-Aha) Ub_{76} was conjugated to me-ADPr-propargyl **2**.
LC-MS (A): Rt = 2.22 min., ESI MS+ (amu) calc: 9483.44 found 9482.60. HRMS: $[C_{412}H_{677}N_{113}O_{136}P_2S + 7H]^{7+}$: found 1355.7113, calc. 1355.7059, $[C_{412}H_{677}N_{113}O_{136}P_2S + 8H]^{8+}$: found 1186.3683, calc. 1186.3687, $[C_{412}H_{677}N_{113}O_{136}P_2S + 9H]^{9+}$: found 1054.6621, calc. 1054.6619, $[C_{412}H_{677}N_{113}O_{136}P_2S + 10H]^{10+}$: found 949.3010, calc. 949.2965, $[C_{412}H_{677}N_{113}O_{136}P_2S + 11H]^{11+}$: found 863.0909, calc. 863.0884,

$[C_{412}H_{677}N_{113}O_{136}P_2S + 12H]^{12+}$: found 791.2516, calc. 791.2484, $[C_{412}H_{677}N_{113}O_{136}P_2S + 13H]^{13+}$: found 730.4630, calc. 730.4606.

Biotin-PEG2-^{triazole42}Ub^{Pr} - Compound (6)

A similar protocol as for compound **4** was used, where Biotin-PEG2-(R42-Aha) Ub_{76} was conjugated to Pr-propargyl **3**.
LC-MS (A): Rt = 2.00 min., ESI MS+ (amu) calc: 9156.21 found 9156.60. HRMS: $[C_{401}H_{663}N_{108}O_{131}PS + 6H]^{6+}$: found 1526.9639, calc. 1526.9767, $[C_{401}H_{663}N_{108}O_{131}PS + 7H]^{7+}$: found 1308.9801, calc. 1308.9812, $[C_{401}H_{663}N_{108}O_{131}PS + 8H]^{8+}$: found 1145.4751, calc. 1145.4845, $[C_{401}H_{663}N_{108}O_{131}PS + 9H]^{9+}$: found 1018.3185, calc. 1018.3204, $[C_{401}H_{663}N_{108}O_{131}PS + 10H]^{10+}$: found 916.5827, calc. 916.5892, $[C_{401}H_{663}N_{108}O_{131}PS + 11H]^{11+}$: found 833.3474, calc. 833.3545, $[C_{401}H_{663}N_{108}O_{131}PS + 12H]^{12+}$: found 763.9880, calc. 763.9923, $[C_{401}H_{663}N_{108}O_{131}PS + 13H]^{13+}$: found 705.2926, calc. 705.3011.

Biotin-PEG2-^{triazole54}Ubme-^{ADPr}, Biotin-PEG2-^{triazole72}Ubme-^{ADPr}, Biotin-PEG2-^{triazole74}Ub$^{me\text{-}ADPr}$

Ub conjugates carrying the me-ADPr modification on positions 54, 72 and 74, respectively, were prepared using the protocol described for conjugate **(5)**.

Biotin-PEG2-^{triazole54}Ub^{Pr}, Biotin-PEG2-^{triazole72}Ub^{Pr}, Biotin-PEG2-^{triazole74}Ub^{Pr}

Ub conjugates carrying the mPr modification on positions 54, 72 and 74, respectively, were prepared using the protocol described for conjugate **(6)**.

References

1. Shin, D., et al., *Regulation of Phosphoribosyl-Linked Serine Ubiquitination by Deubiquitinases DupA and DupB.* Molecular Cell, 2020. **77**(1): p. 164-179.e6.
2. El Oualid, F., et al., *Chemical synthesis of ubiquitin, ubiquitin-based probes, and diubiquitin.* Angew Chem Int Ed Engl, 2010. **49**(52): p. 10149-53.
3. Mulder, M.P.C., et al., *A cascading activity-based probe sequentially targets E1–E2–E3 ubiquitin enzymes.* Nature Chemical Biology, 2016. **12**(7): p. 523-530.
4. Gerlach M., M.U., Weiss S. M.,, *The MX beamlines BL14.1-3 at BESSY II. .* Journal of large-scale research facilities,, 2016. **2, A47.**
5. Kabsch, W., *XDS.* Acta Crystallogr D Biol Crystallogr, 2010. **66**(Pt 2): p. 125-32.
6. Liebschner, D., et al., *Macromolecular structure determination using X-rays, neutrons and electrons: recent developments in Phenix.* Acta Crystallogr D Struct Biol, 2019. **75**(Pt 10): p. 861-877.

7. Pfaffe, M. and R. Mahrwald, *Direct Glycosylation of Unprotected and Unactivated Carbohydrates under Mild Conditions.* Organic Letters, 2012. **14**(3): p. 792-795.
8. Liu, Q., et al., *A General Approach Towards Triazole-Linked Adenosine Diphosphate Ribosylated Peptides and Proteins.* Angewandte Chemie International Edition, 2018. **57**(6): p. 1659-1662.
9. Engelsma, S.B., et al., *Combined Phosphoramidite-Phosphodiester Reagents for the Synthesis of Methylene Bisphosphonates.* 2017. **56**(11): p. 2955-2959.

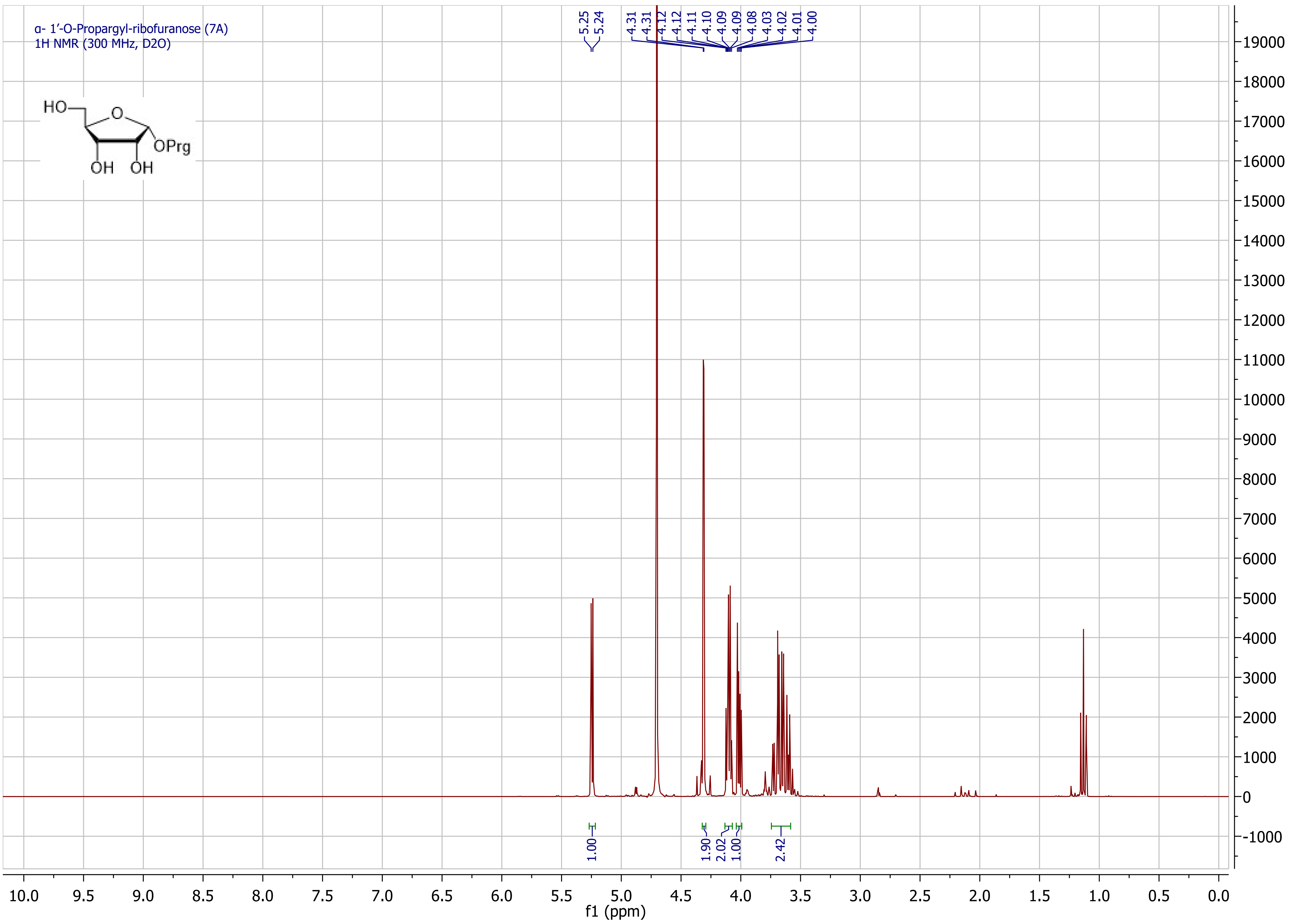
α- 1′-O-Propargyl-ribofuranose (7A)
1H NMR (300 MHz, D2O)
HO
O
OPrg
OH
OH
5.25
5.24
4.31
4.31
4.12
4.12
4.11
4.10
4.09
4.09
4.08
4.03
4.02
4.01
4.00
1.00
1.90
2.02
1.00
2.42
f1 (ppm)

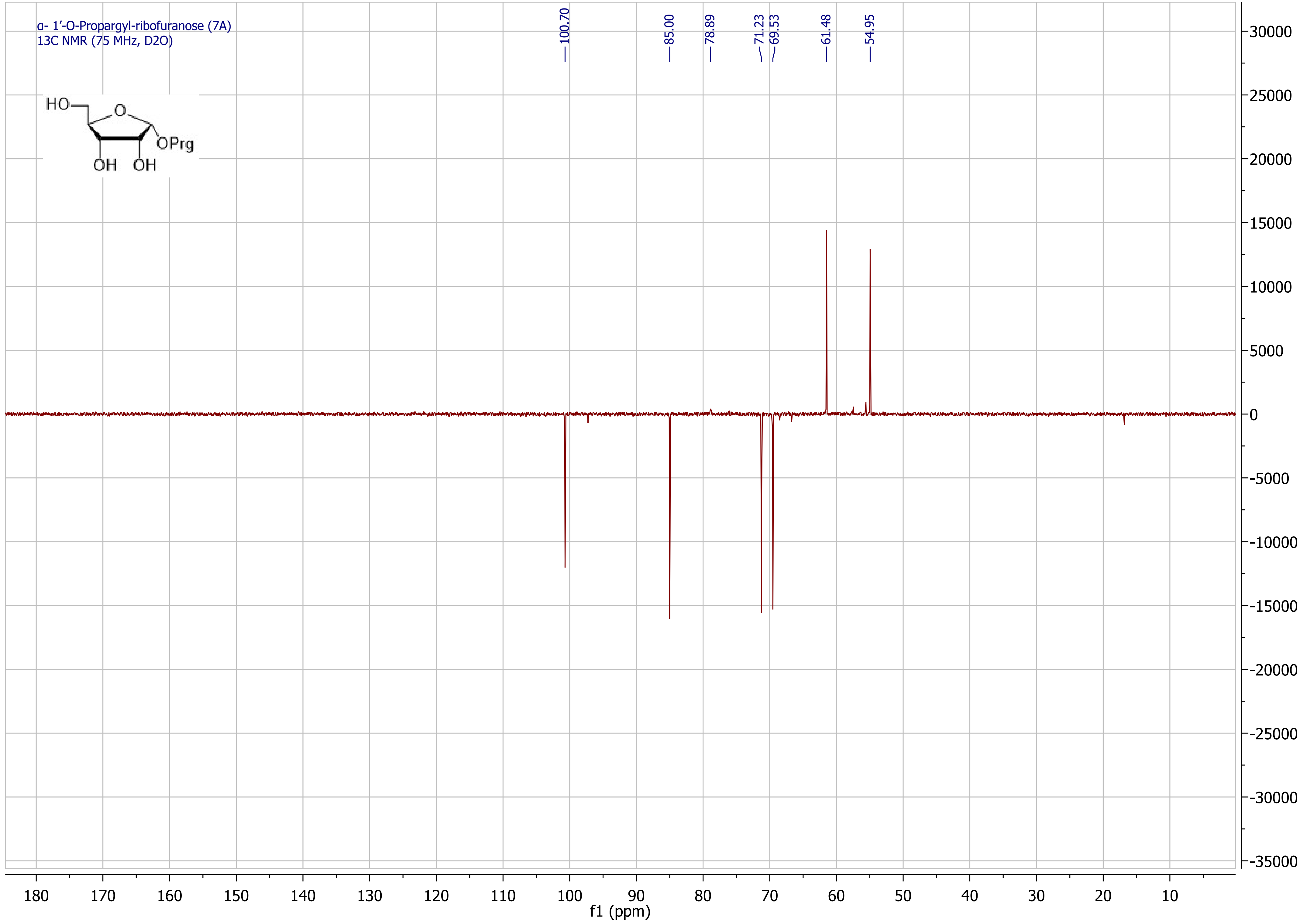

α- 1'-O-Propargyl-ribofuranose (7A)
13C NMR (75 MHz, D2O)
HO
O
OPrg
OH
OH
100.70
85.00
78.89
71.23
69.53
61.48
54.95
30000
25000
20000
15000
10000
5000
0
-5000
-10000
-15000
-20000
-25000
-30000
-35000
180
170
160
150
140
130
120
110
100
90
80
70
60
50
40
30
20
10
f1 (ppm)

β- 1'-O-Propargyl-ribofuranose (7B)
1H NMR (300 MHz, D2O)

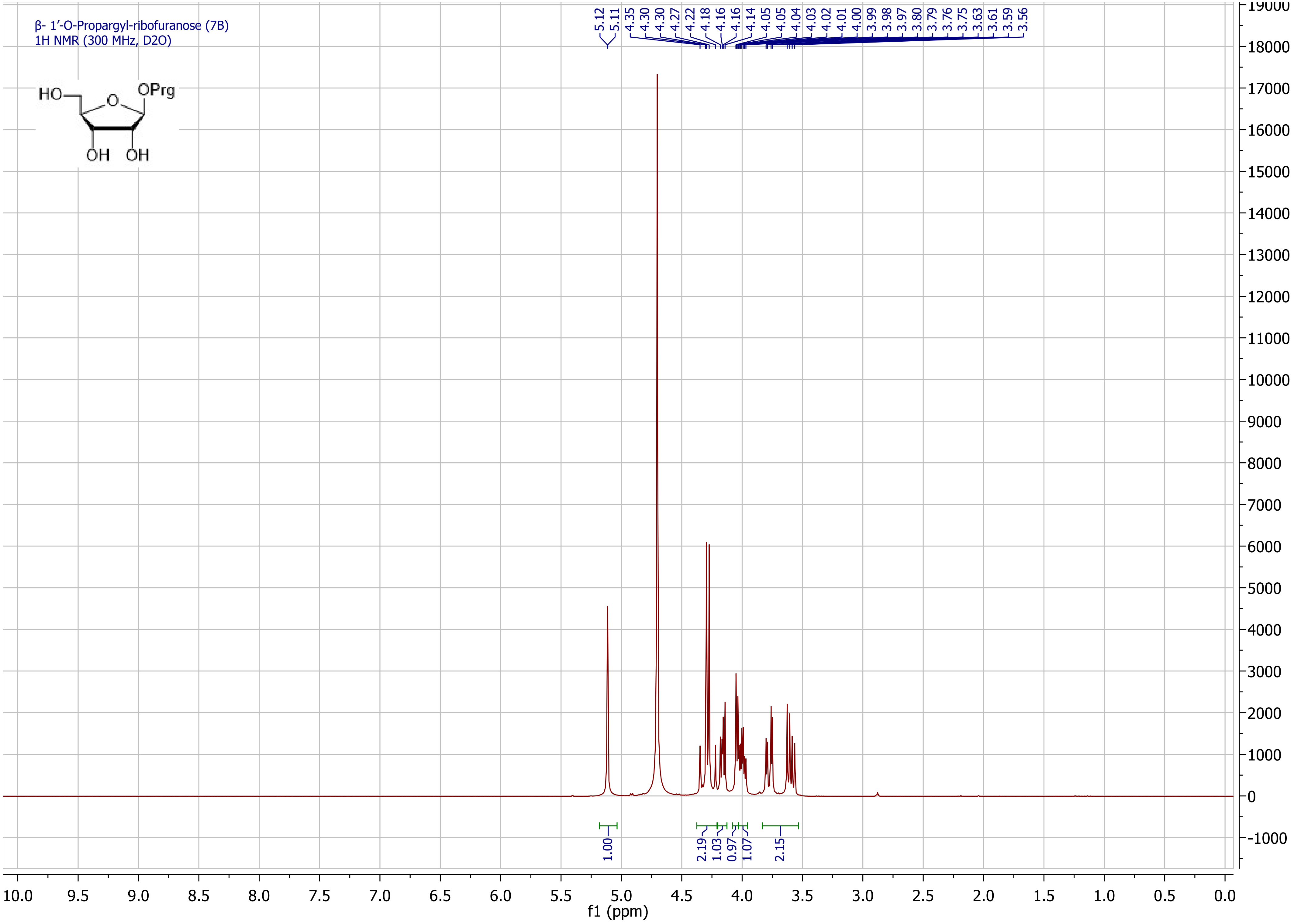

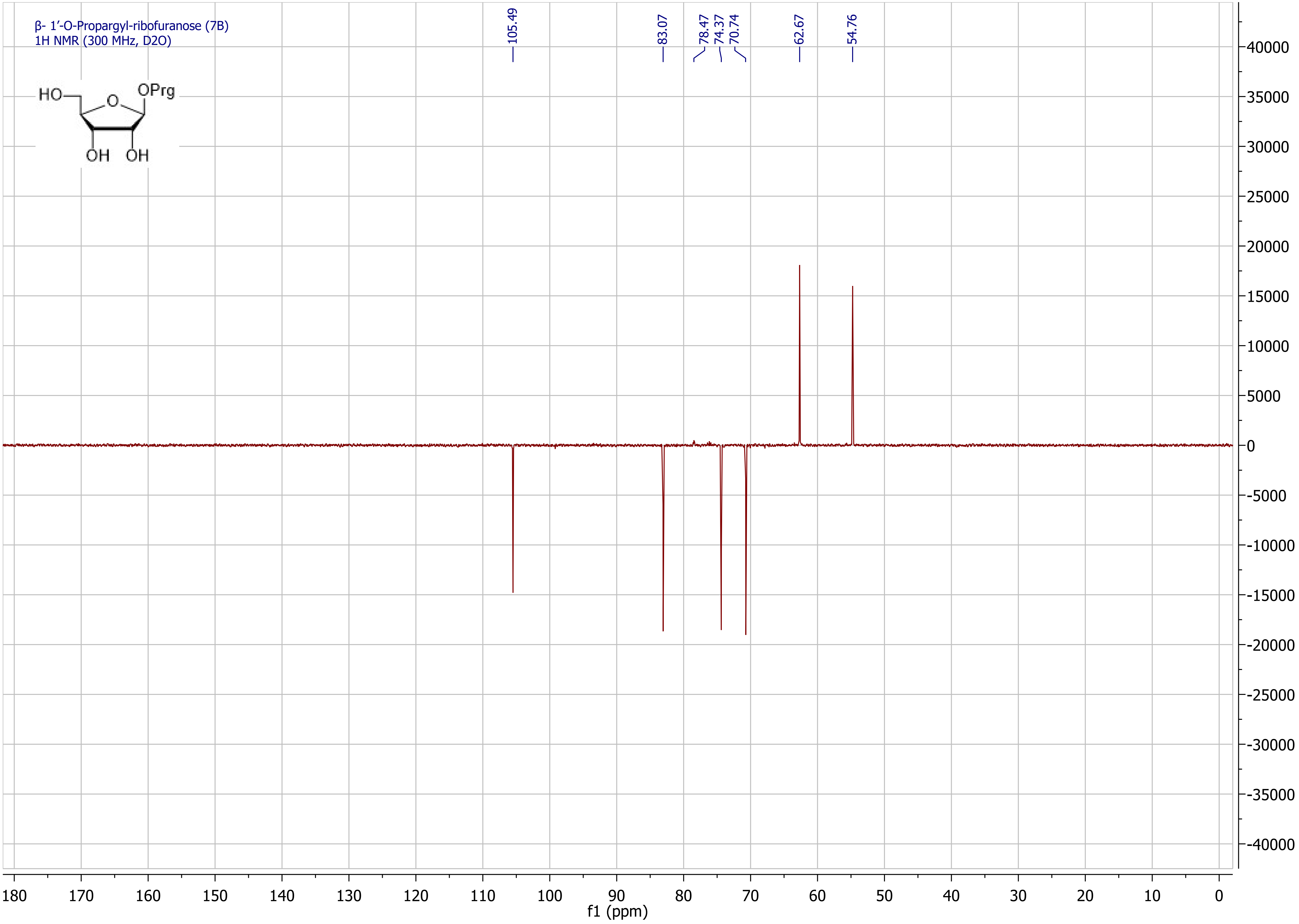
β- 1'-O-Propargyl-ribofuranose (7B)
1H NMR (300 MHz, D2O)
HO
O
OPrg
OH OH
105.49
83.07
78.47
74.37
70.74
62.67
54.76
40000
35000
30000
25000
20000
15000
10000
5000
0
-5000
-10000
-15000
-20000
-25000
-30000
-35000
-40000
180
170
160
150
140
130
120
110
100
90
80
70
60
50
40
30
20
10
0
f1 (ppm)

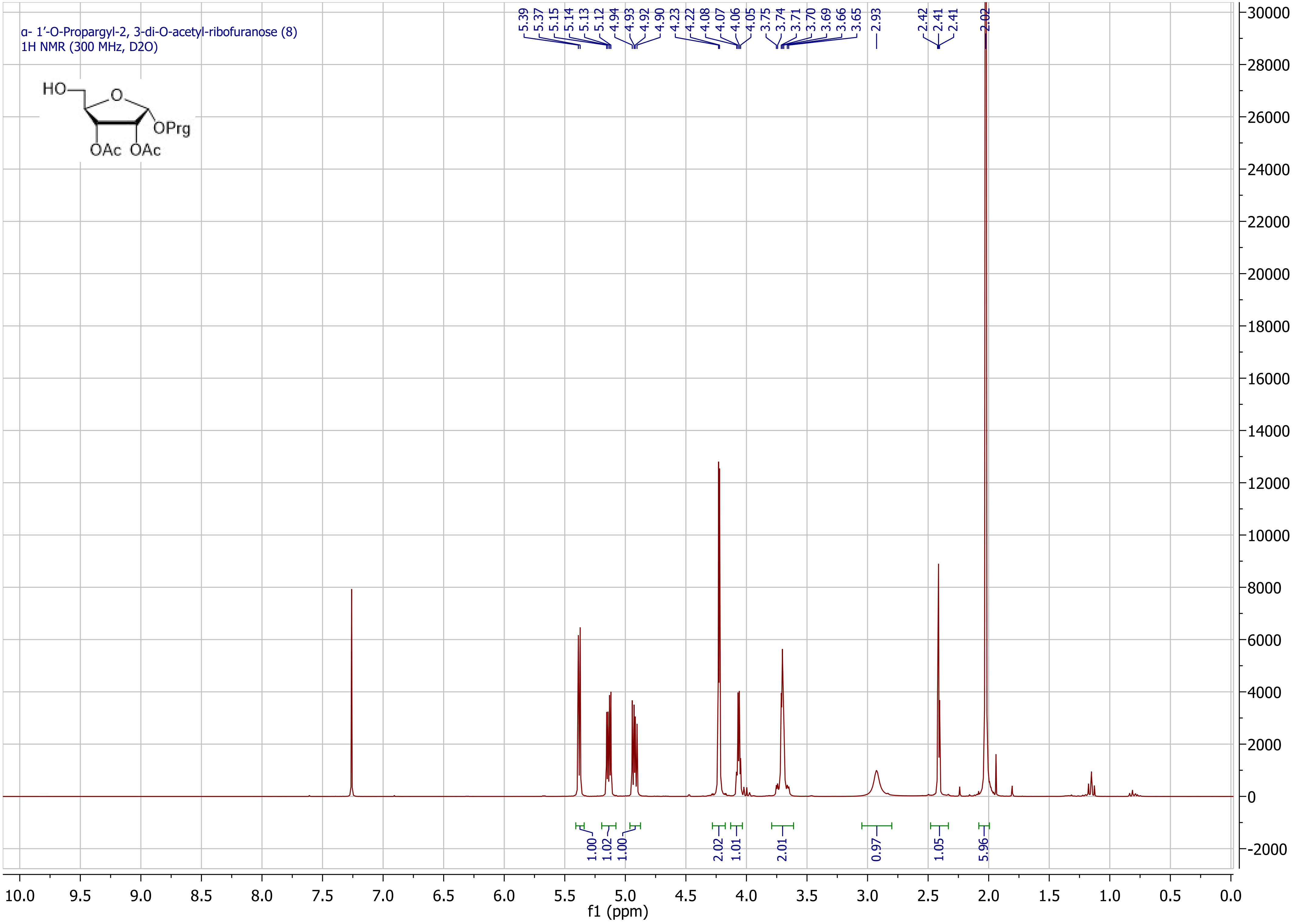

α- 1'-O-Propargyl-2, 3-di-O-acetyl-ribofuranose (8)
1H NMR (300 MHz, D2O)
HO
O
OPrg
OAc OAc
5.39
5.37
5.15
5.14
5.13
5.12
4.94
4.93
4.92
4.90
4.23
4.22
4.08
4.07
4.06
4.05
3.75
3.74
3.71
3.70
3.69
3.66
3.65
2.93
2.42
2.41
2.41
2.02
1.00
1.02
1.00
2.02
1.01
2.01
0.97
1.05
5.96
f1 (ppm)

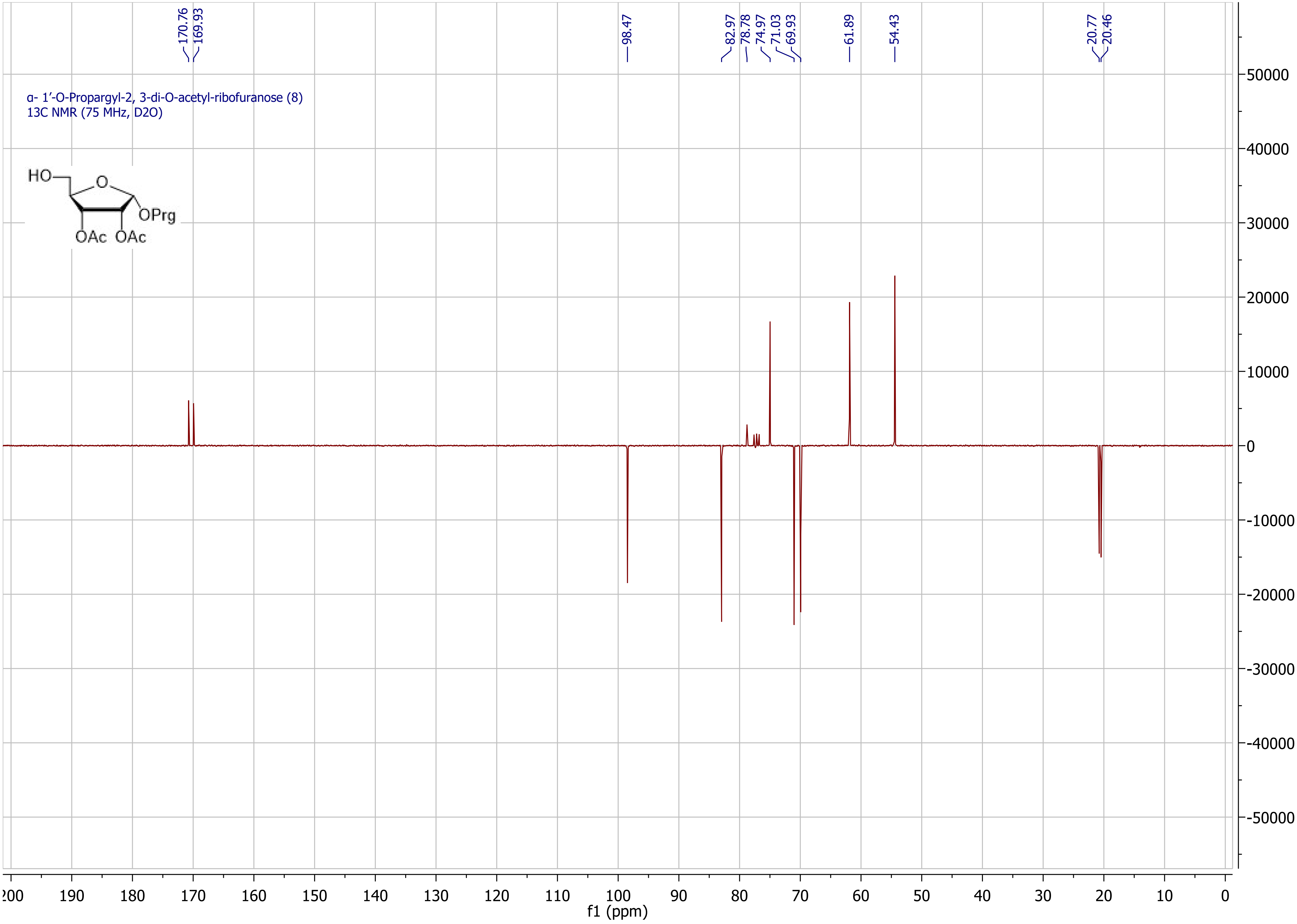

α- 1′-O-Propargyl-2, 3-di-O-acetyl-ribofuranose (8)
13C NMR (75 MHz, D2O)
HO
O
OPrg
OAc OAc
170.76
169.93
98.47
82.97
78.78
74.97
71.03
69.93
61.89
54.43
20.77
20.46
50000
40000
30000
20000
10000
0
-10000
-20000
-30000
-40000
-50000
200 190 180 170 160 150 140 130 120 110 100 90 80 70 60 50 40 30 20 10 0
f1 (ppm)

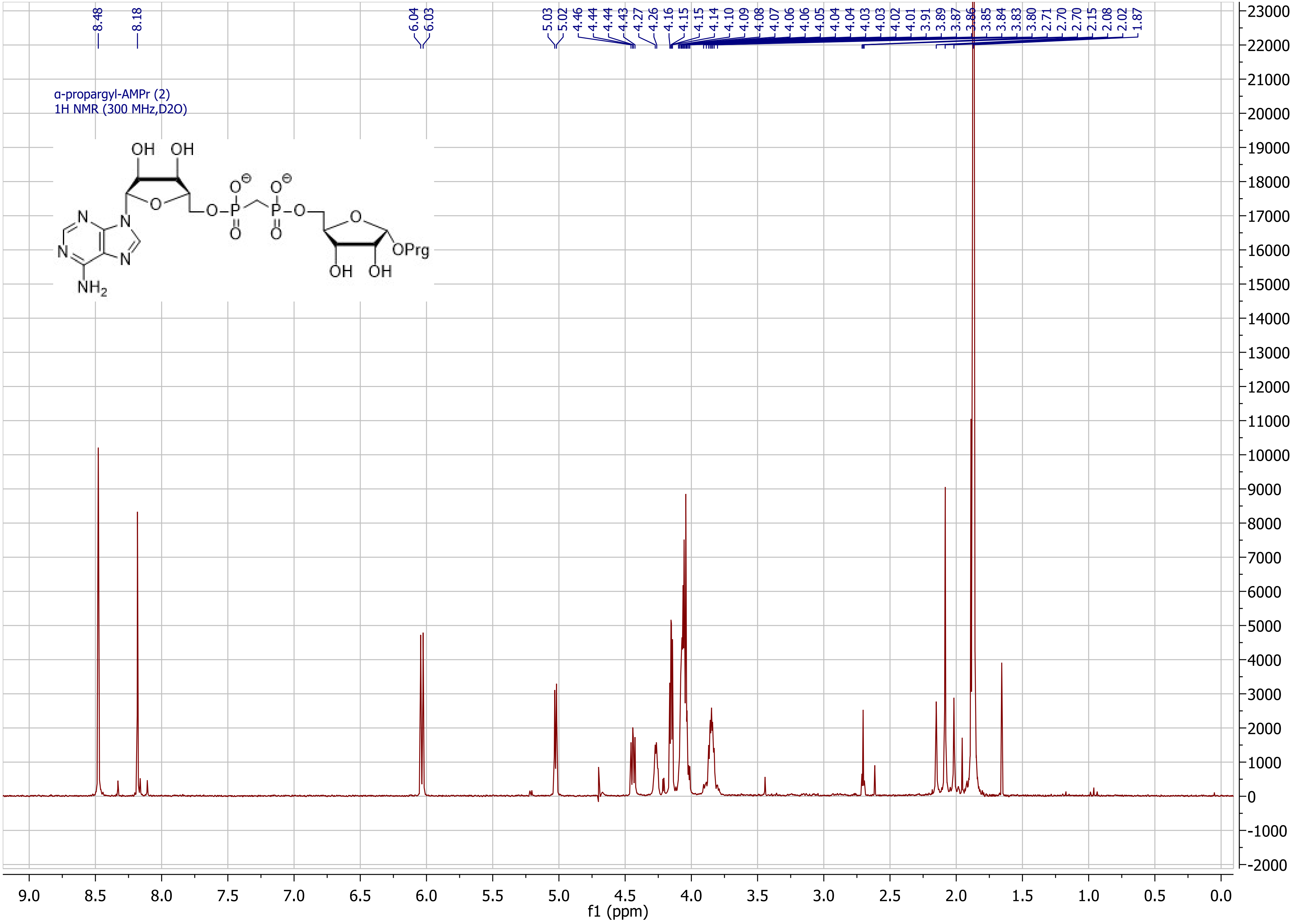
α-propargyl-AMPr (2)
1H NMR (300 MHz,D2O)
OPrg
NH2
8.48
8.18
6.04
6.03
5.03
5.02
4.46
4.44
4.44
4.43
4.27
4.26
4.16
4.15
4.15
4.14
4.10
4.09
4.08
4.07
4.06
4.06
4.05
4.04
4.04
4.03
4.03
4.02
4.01
3.91
3.89
3.87
3.86
3.85
3.84
3.83
3.80
2.71
2.70
2.70
2.15
2.08
2.02
1.87
f1 (ppm)

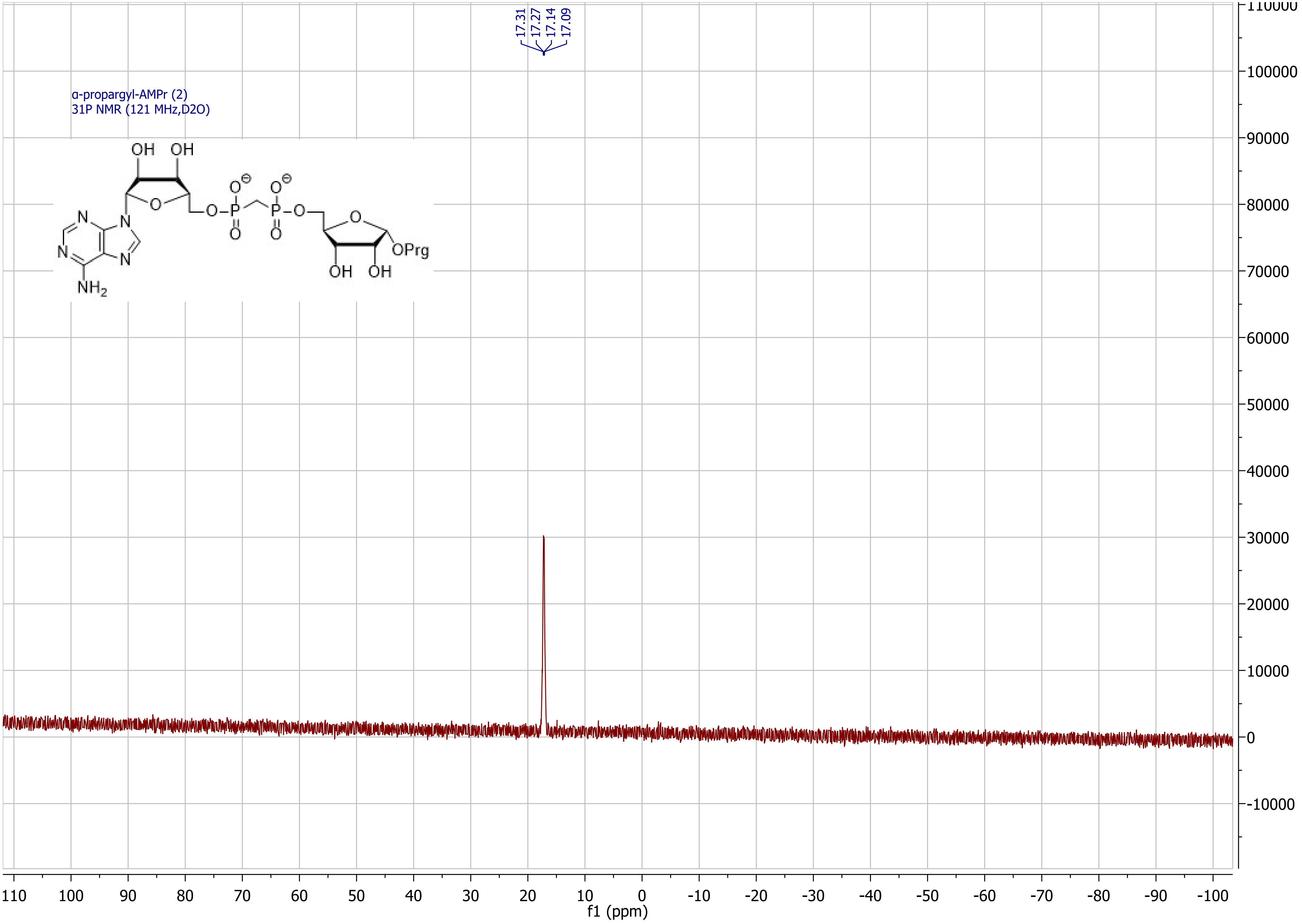
α-propargyl-AMPr (2)
31P NMR (121 MHz,D2O)
17.31
17.27
17.14
17.09
OH OH
O
O
P
P
N
N
N
N
NH2
OPrg
OH OH
110000
100000
90000
80000
70000
60000
50000
40000
30000
20000
10000
0
-10000
110 100 90 80 70 60 50 40 30 20 10 0 -10 -20 -30 -40 -50 -60 -70 -80 -90 -100
f1 (ppm)

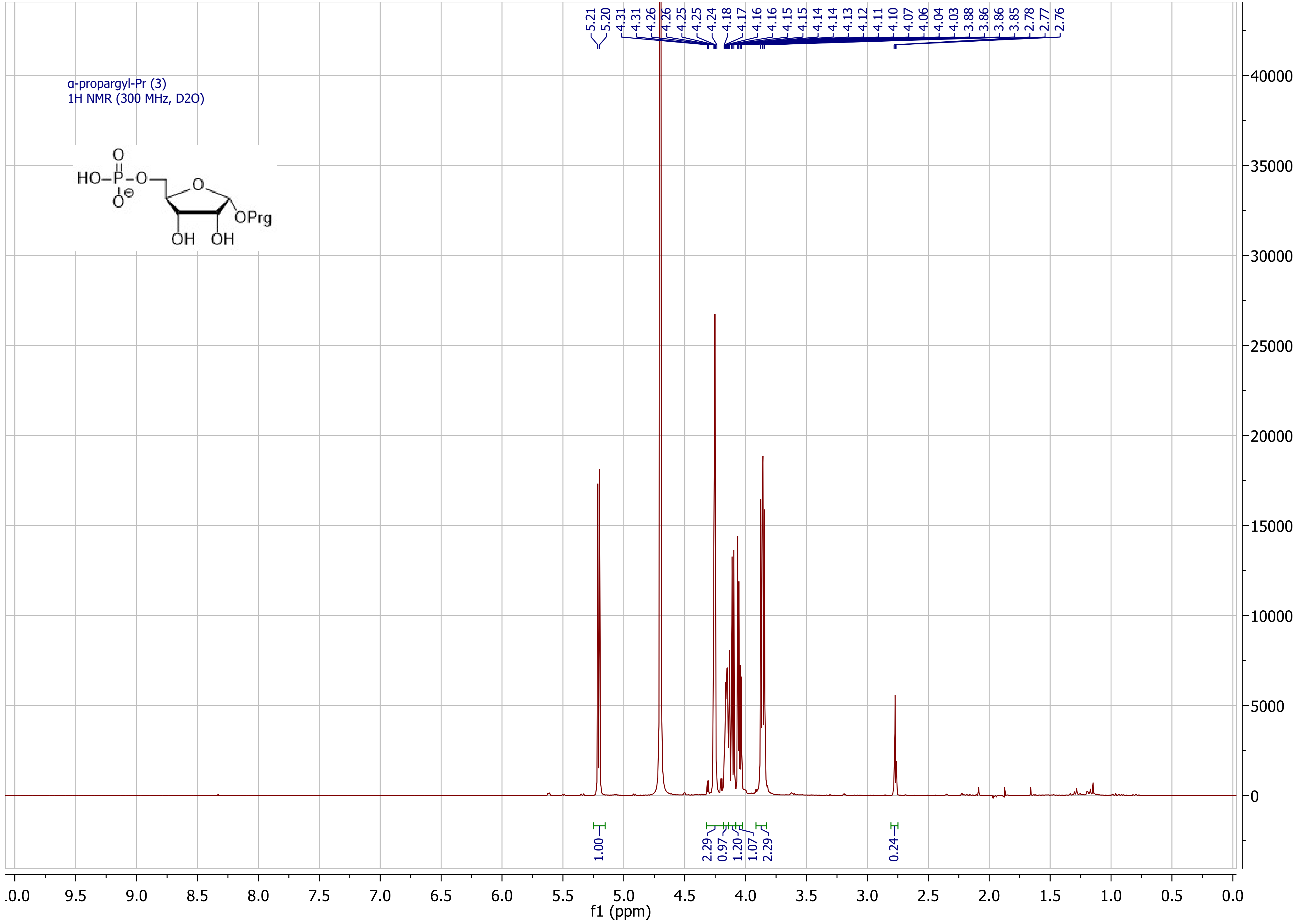
α-propargyl-Pr (3)
1H NMR (300 MHz, D2O)
HO
P
O
O
O
O
OPrg
OH
OH
5.21
5.20
4.31
4.26
4.26
4.25
4.25
4.24
4.18
4.17
4.16
4.16
4.15
4.15
4.14
4.14
4.13
4.12
4.11
4.10
4.07
4.06
4.04
4.03
3.88
3.86
3.86
3.85
2.78
2.77
2.76
1.00
2.29
0.97
1.20
1.07
2.29
0.24
f1 (ppm)
.0.0
9.5
9.0
8.5
8.0
7.5
7.0
6.5
6.0
5.5
5.0
4.5
4.0
3.5
3.0
2.5
2.0
1.5
1.0
0.5
0.0
40000
35000
30000
25000
20000
15000
10000
5000
0

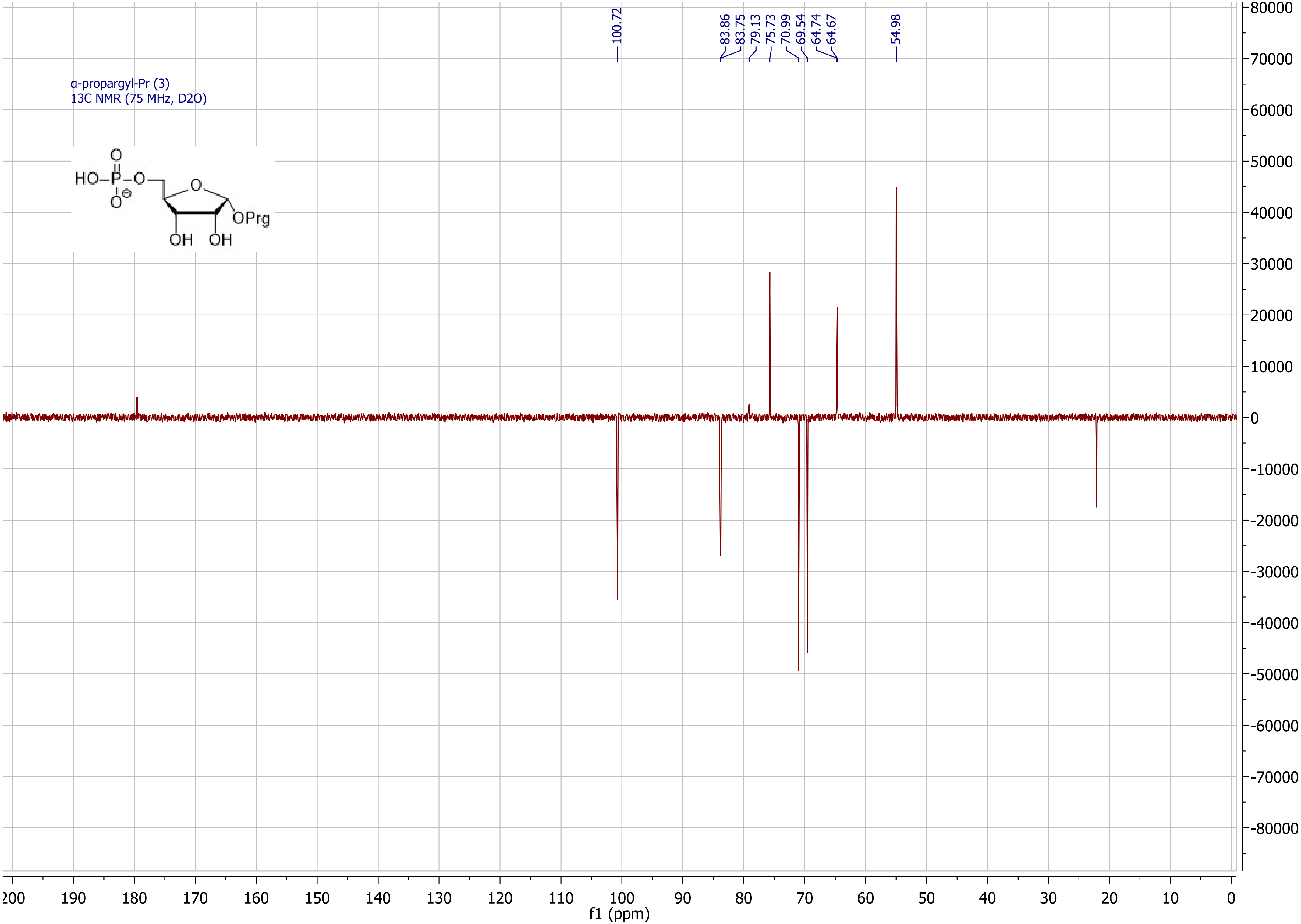
α-propargyl-Pr (3)
13C NMR (75 MHz, D2O)
HO–P–O–
O
O⊖
O
OPrg
OH
OH
100.72
83.86
83.75
79.13
75.73
70.99
69.54
64.74
64.67
54.98
f1 (ppm)

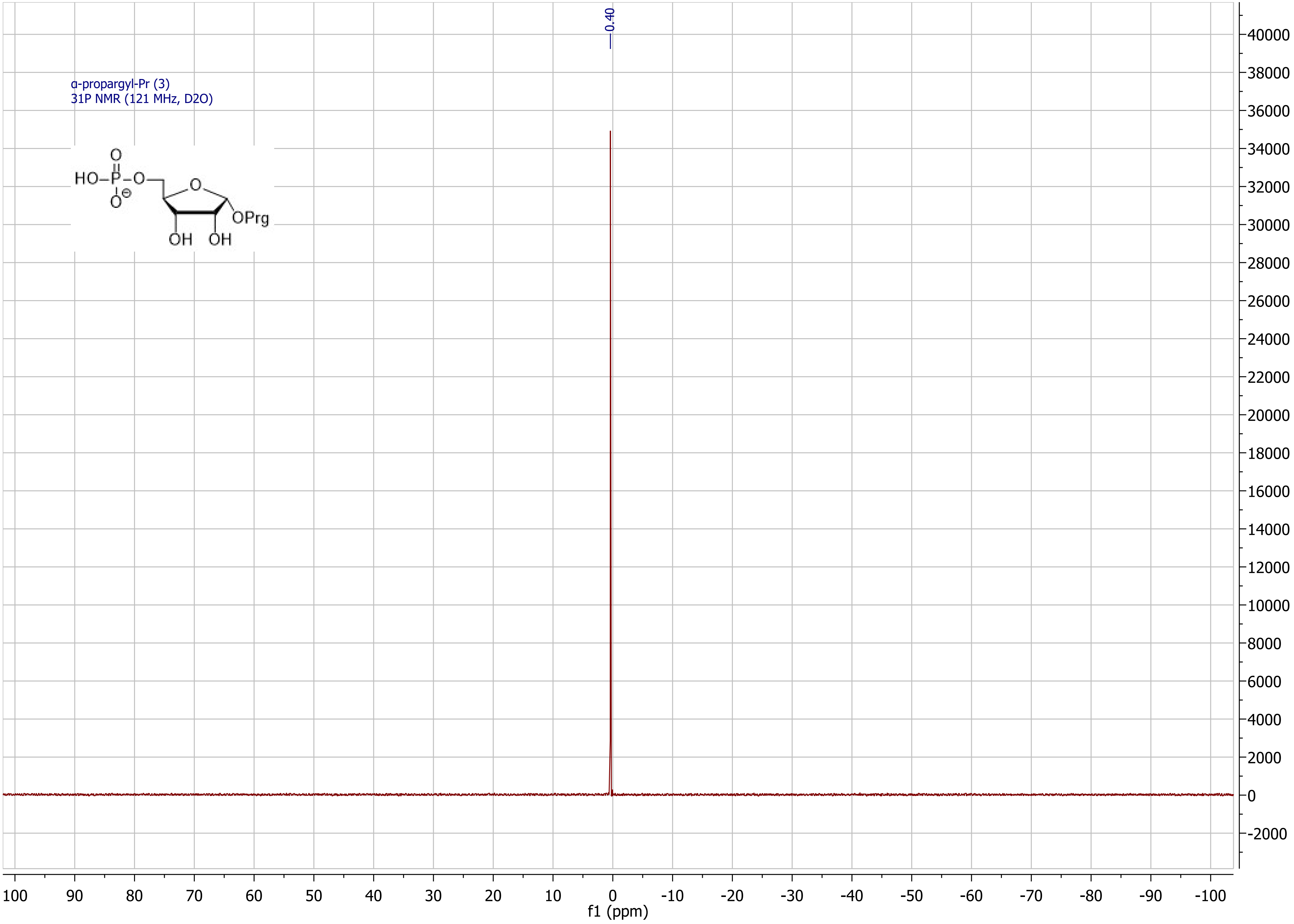

α-propargyl-Pr (3)
31P NMR (121 MHz, D2O)
HO–P–O
O
O⊖
OPrg
OH OH
0.40
f1 (ppm)
100 90 80 70 60 50 40 30 20 10 0 -10 -20 -30 -40 -50 -60 -70 -80 -90 -100
40000 38000 36000 34000 32000 30000 28000 26000 24000 22000 20000 18000 16000 14000 12000 10000 8000 6000 4000 2000 0 -2000
